# Comprehensive Epigenome-Wide Profiling Reveals Distinctive DNA Methylation Signatures and Potential Prognostic Biomarkers in Mexican Pediatric B-ALL

**DOI:** 10.3390/ijms262110261

**Published:** 2025-10-22

**Authors:** Alan Alberto Fong-López, Juan Carlos Núñez-Enríquez, Vilma Carolina Bekker-Méndez, Janet Flores-Lujano, Minerva Mata-Rocha, Elva Jiménez-Hernández, Mónica Patricia Ortíz-Maganda, Francisco Xavier Guerra-Castillo, Aurora Medina-Sanson, Jorge Alfonso Martín-Trejo, José Gabriel Peñaloza-González, Martha Margarita Velázquez-Aviña, José Refugio Torres-Nava, Rosa Martha Espinosa-Elizondo, María Luisa Pérez-Saldívar, Luz Victoria Flores-Villegas, Laura Elisa Merino-Pasaye, David Aldebaran Duarte-Rodríguez, Omar Alejandro Sepúlveda-Robles, Georgina Jiménez-Morales, Haydeé Rosas-Vargas, Jorge Meléndez-Zajgla, Eva Ramón-Gallegos, Juan Manuel Mejía-Aranguré, Silvia Jiménez-Morales

**Affiliations:** 1 Laboratorio de Innovación y Medicina de Precisión, Núcleo “A”, Instituto Nacional de Medicina Genómica (INMEGEN), Mexico City 14610, Mexico; alanalbertofong@gmail.com (A.A.F.-L.); gyna.uamy@gmail.com (G.J.-M.); 2 Programa de Doctorado en Biomedicina y Biotecnología Molecular, Escuela Nacional de Ciencias Biológicas, Instituto Politécnico Nacional, Mexico City 07738, Mexico; 3 Laboratorio de Citopatología Ambiental, Departamento de Morfología, Escuela Nacional de Ciencias Biológicas, Instituto Politécnico Nacional, Campus Zacatenco, Mexico City 07738, Mexico; eramong@ipn.mx; 4 Facultad de Ciencias Químico-Biológicas, Universidad Autónoma de Sinaloa, Culiacán Rosales 80030, Mexico; 5 División de Investigación en Salud, Hospital de Pediatría “Dr. Silvestre Frenk Freund”, Centro Médico Nacional “Siglo XXI”, Instituto Mexicano del Seguro Social (IMSS), Mexico City 06720, Mexico; jcarlos_nu@hotmail.com (J.C.N.-E.); jorgemartint@gmail.com (J.A.M.-T.); 6 Unidad de Investigación Biomédica en Inmunología e Infectología, Hospital de Infectología “Dr. Daniel Méndez Hernández”, Centro Médico Nacional “La Raza”, Instituto Mexicano del Seguro Social (IMSS), Mexico City 02990, Mexico; bekkermendez@yahoo.com (V.C.B.-M.); moniomaganda@gmail.com (M.P.O.-M.); pacoxguerra@gmail.com (F.X.G.-C.); 7 Unidad de Investigación Médica en Epidemiología Clínica, Hospital de Pediatría “Dr. Silvestre Frenk Freund”, Centro Médico Nacional “Siglo XXI”, Instituto Mexicano del Seguro Social (IMSS), Mexico City 06720, Mexico; janetflores22@yahoo.com.mx (J.F.-L.); maria_luisa_2000_mx@yahoo.com (M.L.P.-S.); 8 Unidad de Investigación Médica en Genética Humana, Hospital de Pediatría “Dr. Silvestre Frenk Freund”, Centro Médico Nacional “Siglo XXI”, Instituto Mexicano del Seguro Social (IMSS), Mexico City 06720, Mexico; mine_mr@hotmail.com (M.M.-R.); sero__82@hotmail.com (O.A.S.-R.); hayrov@gmail.com (H.R.-V.); 9 Servicio de Hematología Pediátrica, Secretaría de Salud de la Ciudad de México (SSCDMX), Hospital Pediátrico de Moctezuma, Mexico City 15530, Mexico; elvajimenez@yahoo.com (E.J.-H.); torresoncoped@live.com.mx (J.R.T.-N.); 10 Departamento de Oncología, Hospital Infantil de México Federico Gómez, Mexico City 06720, Mexico; auroramedina@aol.com.mx; 11 Servicio de Onco-Pediatría, Hospital Juárez de México, Mexico City 07760, Mexico; penaloza_6@yahoo.es (J.G.P.-G.); m_mvelazquez@yahoo.com.mx (M.M.V.-A.); 12 Servicio de Hematología Pediátrica, Secretaría de Salud (SS), Hospital General de México, Mexico City 06720, Mexico; rmespinosa1605@hotmail.com; 13 Servicio de Hematología Pediátrica, Centro Médico Nacional “20 de Noviembre”, Instituto de Seguridad Social al Servicio de los Trabajadores del Estado (ISSSTE), Mexico City 03104, Mexico; victoriabanco@yahoo.com.mx (L.V.F.-V.); sketch0712@gmail.com (L.E.M.-P.); 14 División de Desarrollo de la Investigación en Salud, Coordinación de Investigación en Salud, Centro Médico Nacional “Siglo XXI”, Instituto Mexicano del Seguro Social (IMSS), Mexico City 06720, Mexico; david.duarte@imss.gob.mx; 15 Laboratorio de Genómica Funcional del Cáncer, Instituto Nacional de Medicina Genómica (INMEGEN), Mexico City 14610, Mexico; jmelendez@inmegen.gob.mx; 16 Facultad de Medicina, Universidad Nacional Autónoma de México, Mexico City 04360, Mexico

**Keywords:** acute lymphoblastic leukemia, DNA methylation, relapse, *MAD1L1*, *RNH1*, *FBXL22*, Mexican pediatric population

## Abstract

Acute lymphoblastic leukemia (ALL) is the most common childhood cancer. In Mexico, its higher incidence and lower survival suggest a role for epigenetic factors like DNA methylation (DNAme). We conducted an epigenome-wide association study (EWAS) to define the methylation landscape and identify the profiles associated with ALL and relapse. Bone marrow or peripheral blood samples from pediatric ALL patients at diagnosis and controls without ALL were analyzed using an Infinium MethylationEPIC v2.0 array. Differential methylation was assessed using the ChAMP package. We identified a significant hypermethylated profile in ALL patients compared to controls. Probes in *MAD1L1* and *RPTOR* contained the most differentially methylated CpG sites. Key affected pathways included proliferation, neurotransmission, and neuronal signaling. Survival analysis revealed that hypomethylation of four specific CpGs—cg01052776 (*RNH1*), cg20747787, cg05001671, and cg01767116 (*FBXL22*)—was significantly associated with an increased risk of relapse, highlighting their potential as prognostic biomarkers. This study underscores the importance of epigenetic mechanisms in pediatric ALL.

## 1. Introduction

Acute lymphoblastic leukemia (ALL) is the most common pediatric malignancy, accounting for approximately 25% of all childhood cancer diagnoses [1,2]. It is characterized by the uncontrolled proliferation of lymphoid blasts and exhibits significant clinical and molecular heterogeneity, which complicates its therapeutic management. Although ALL displays a consistent age pattern across ethnic groups, variable incidences have been reported among them. In Mexico, similarly to other Latin American nations (Mexico: 5.3; Manaus: 5.7; Costa Rica: 5.2 cases per 100,000 children), the incidence of this malignancy is higher than in countries such as United States (4.1 cases per 100,000 children) or other Caucasian populations [3,4,5], and, according to several reports, its incidence is increasing [2,4,6,7,8]. Moreover, the five-year survival rate is considerably lower in Mexico (66%), even worse than other Latin American populations and developed countries, which is currently around 85–90% [5,6,8,9,10,11,12]. Actually, more than 95% five-year survival rates have been reported by groups such as the Nordic Society of Pediatric Hematology and Oncology (NOPHO) and the Dutch Childhood Oncology Group (DCOG) [13,14,15] and countries such as Finland and Qatar [16,17], and even 100% in China for patients with *TCF3*::*PBX1* rearrangement [17].

In addition to disparities in ALL incidence and overall survival, differences in clinical presentation and molecular features have also been reported among various populations. For instance, Mexican patients display higher relapse rates and a lower prevalence of favorable prognostic fusion genes, such as *ETV6*::*RUNX1*, compared to Caucasian populations (26.2% vs. 10–15%, respectively) [3,18,19,20,21]. Relapse in ALL cases was found in 26.2% of ALL children from Mexico, while it was found in 10–15% in Caucasian from the United States [10] and 2–4% in Nordic Caucasian populations [15,21]. Regarding good prognosis biomarkers, studies have shown that *ETV6::RUNX1* is present in 7–13% of Mexican cases, but around 15–25% in Caucasian, Black, and Asian populations [3,5,13,14,22,23]. Significant differences have also been reported in other molecular features such as *TCF3::PBX1*, which displays frequencies between 15 and 25% in Asian-, African-, and Caucasian-descendant populations but 7.1–11.5% in Mexicans [3,17,20,22,23,24].

These differences have been partially attributed to the influence of specific genetic ancestry backgrounds and epigenetic factors, such as DNA methylation (DNAme), which is recognized as an early and fundamental event in cell transformation and cancer development [24,25,26]. Genetic causes of ALL have been addressed by multiples studies, discovering racial/ethnic disparities in the frequencies of specific genetic variants linked to ALL and treatment outcome [5,26,27] and uncovering new molecular subtypes and subtype-specific alterations with differential prognostic impacts [13,28,29,30]. Nevertheless, epigenomic studies are still sparse, and most of them have focused on European-descent populations [30,31,32,33,34,35]; ethnic groups with different genetic ancestries and environmental exposures, such as Latin American, remain poorly explored [36,37].

DNAme is a chemical modification process in which a methyl group is added to cytosine residues at CpG dinucleotide sites, regulating gene expression and maintaining genome stability, as well as influencing cell fate. Aberrant DNAme, mainly in CpG islands located within gene regulatory elements (e.g., promoter regions), often leads to the silencing of cancer-controlling genes such as tumor suppressor genes. Conversely, hypomethylation may activate the expression of proto-oncogenes, thereby promoting uncontrolled cell proliferation [38]. As in other types of cancers, oncogenes, tumor suppressor genes, and DNA repair genes are frequently affected by abnormal methylation in ALL [21,39]. In addition, genomic and epigenomic studies have shown that, in contrast to adult cancer, most of the altered genes in ALL are epigenetic regulators, which can conduct alterations in DNAme patterns [5,26,34,40,41]. Genes such as *IKZF1*, *CDKN2B*, *TET2*, *CYP1B1*, *PTPN6*, *HOPX*, *SMIM24*, and *PPP1R10*, which play key roles in the regulation of hematopoietic processes, commonly exhibit altered methylation patterns in ALL [5,26,39,41]. Other genes revealed in epigenomic studies to be relevant players in ALL, mainly in Down syndrome ALL, include *TRIM13*, which was identified in newborn dried blood spots obtained from biobanks [39].

Beyond the growing body of evidence supporting the role of DNAme in the pathogenesis of leukemia [42], methylation profiles are also being investigated for their potential use in molecular classification, outcome and chemotherapy resistance prediction, and as novel therapeutic targets [35,43,44]. For example, a wide epigenomic association study conducted by the NOPHO ALL2008 and DCOG ALL10/11 proposed an epigenetics-based classifier for pediatric T-ALL that distinguishes two risk groups of patients based on a CpG island methylator phenotype [45].

In addition, data from paired diagnosis-relapsed samples showed a lower global promoter methylation at diagnosis in contrast to relapse [42], and therapy with decitabine, a DNA methyltransferase inhibitor, restores chemosensitivity in animal models [44]. To identify aberrantly methylated genes in pediatric B-cell ALL (B-ALL) and to evaluate their association with relapse, we conducted an epigenome-wide association study (EWAS) in a prospective cohort of Mexican children.

## 2. Results

### 2.1. Clinical Characteristics of the Analyzed Population

We included 53 pediatric ALL cases, of which 45% (*n* = 24) were male. The median age at diagnosis was 37 months (range: 4–205 months old). The most common fusion gene was *ETV6*::*RUNX1* (*n* = 11, 20%), followed by *TCF3*::*PBX1* (*n* =6, 12%). According to the National Cancer Institute (NCI) criteria, 53% (*n*= 28) of cases were classified as standard risk, while 47% (*n* = 25) were classified as high risk. Based on the most recent WHO classification tumors of hematopoietic and lymphoid tissues (WHO-HEM5) and by incorporating molecular data from tested fusion genes, the distribution of molecular genetic subtypes was as follows: 20% of cases were classified as *ETV6*::*RUNX1*-rearranged, 12% as *TCF3*::*PBX1*-rearranged, 4% as *BCR*::*ABL1*-rearranged, and 2% as *KMT2A*-rearranged.

Non-ALL controls consisted of four individuals aged 0–17 years, 50% female and 50% males, and median age 37 months (range: 4–205 months old).

The initial cohort included 53 patients; however, 7 of them abandoned treatment before reaching 18 months of follow-up (median follow-up: 1 month; range: 0–15 months), and the remaining (*n* = 46) completed at least 18 months of follow-up after initial treatment. Further analyses were only performed on patients with complete clinical records. Control samples were included only in case–control comparisons. Patients without appropriate follow-up were excluded from relapse and early relapse analyses. Among the remaining group, relapse was observed in 39% (18/46); of these, 61% (*n* = 11) experienced very early relapse (VER) and 39% (*n* = 7) experienced early relapse (ER) (Table 1).

### 2.2. Identification of Differential Methylated CpGs in Acute Lymphoblastic Leukemia

Due to the fact that we obtained PB (*n* = 16) or BM (*n* = 37) from our patients, and because PB cells came from the BM, we first compared their methylation profiles to determine whether PB and BM samples could be combined for subsequent analyses. It is worth mentioning that both BM and PB have >75% of lymphoblasts, ensuring that the captured methylation data was predominantly derived from the leukemic cell population, regardless of tissue source. This comparative analysis revealed 99.06% (6711/719,243 probes) similarities in the CpGs between both tissues, and only 3 of them were detected with Δβ |>20|%. Even when *p*-values were significant, none of these resulted in a differentially methylated site (DMC) (FDR >0.05). Additionally, multidimensional scaling analysis confirmed the absence of systematic clustering by tissue origin (Supplementary Figure S1). Then, a subsequent analysis included all ALL patients.

**Figure 1:**
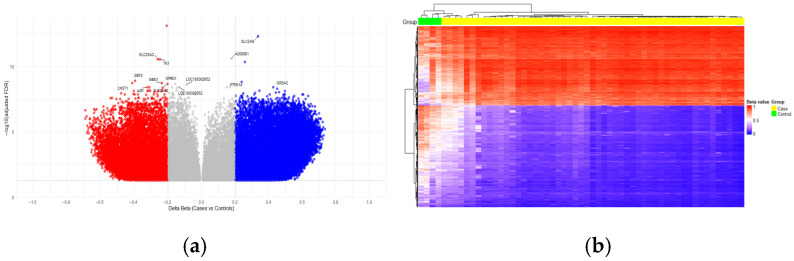
Case–control analysis showing differences in methylation levels between pediatric patients with ALL (yellow) and controls (green). (**a**) Unsupervised hierarchical clustering of differentially methylated CpGs (DMC) based on methylation differences (Δβ) between ALL cases and controls. The heatmap shows a cluster diagram of the top 500 DMCs in ALL patients versus controls. The scale on the right represents the methylation status (red: hypermethylated, blue. hypomethylated). (**b**) The volcano plot displays the DMCs between cases and controls, with FDR-adjusted <0.05 and absolute Δβ (|Δβ|) >0.2. The x-axis shows the methylation difference (Δβ), and the y-axis represents –log10 of the adjusted *p*-value. Hypermethylated (red) and hypomethylated (blue) CpGs are highlighted.

To further identify a methylation signature specific to ALL, a comparative analysis was performed. A total of 37,541 DMCs (hypomethylation 19,101 vs. hypermethylation: 18,440 CpGs) located in 12862 genes were identified at an FDR < 0.05 (Figure 1a, Supplementary Table S1). As expected, several previously reported genes with abnormal methylation in leukemias such as *CPEB1*, *BLK*, *FLT3*, *ZCCHC7*, *EBF1*, *HDACs*, *IKZF1*, *RB1*, *CDK6*, *DOCK5*, *DPP10*, *MUC4*, *JAKs*, *KIT*, *KRAS*, *LINC01013*, *MAD1L1*, *NOTCH1*, and *STATs*, among others, were also detected (Supplementary Table S1). The median absolute difference in methylation levels among these DMCs was 0.40 (range: from 0.20 to 0.73) for hypomethylated and −0.35 (range: from −0.69 to −0.20) for hypermethylation.

Unsupervised-clustering analysis detected the 500 DMCs that define an ALL profile (Figure 1b). The DMCs located in *SLC2A9*, *ADGRD1*, *TK1*, *SLC25A2*, *SBF2*, *PTPN14*, *CHST1*, *MBD1*, *OPRD1*, *LOC100302652*, *OR5A2*, *APP*, *LOC100302652* showed the highest statistical significance (FDR < 4 × 10^−8^).

**Figure 2:**
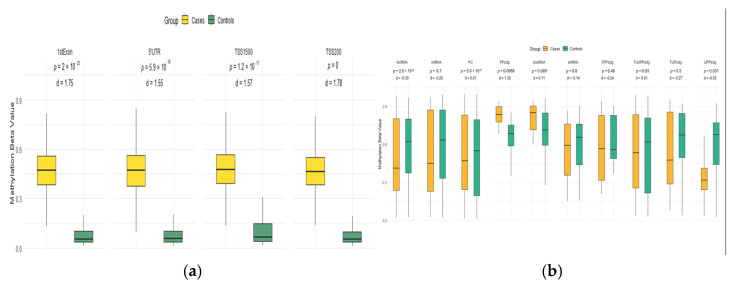
Box plot results of the DNA methylation levels derived from ALL cases are shown on CpG islands of promoter regions (**a**) and on gene biotypes (**b**) in ALL cases (yellow) and controls (green). The line inside each box represents the median of the methylation β values of the samples. The upper and lower edges of the boxes are the whiskers (75th percentiles and 25th, respectively), while upper and lower lines outside the boxes are the error bars. *p*-values < 0.05 (Mann–Whitney test) mean that the differences in DNA methylation between ALL patients and controls are statistically significant. 1stExon: first exon; 5′UTR: 5′ untranslated region; TSS: transcriptional start site; PC: protein coding; lncRNA: long non-coding RNA; miRNA: micro-RNA; TUPsdg: transcribed unitary pseudogene; TPPsdg: transcribed processed pseudogene; TUnPPsdg: transcribed unprocessed pseudogene; UPPsdg: unprocessed pseudogene; PPsdg: processed pseudogene.

Based on the knowledge that DMCs can affect the expression of relevant genes involved in the pathogenesis of ALL, we explored CpGs located into the TSS200, TSS1500, 1stExon, 5′UTR regions, and body genes. Among all DMCs identified, 11,226 were positioned in promoter regions, of which 3264 were in 5′ untranslated regions (5′UTRs), 1,413 in the first exons, 2657 in the region 200bp before the transcription start site (TSS200), and 3,893 in the region 1500bp before the transcription start site (TSS1500). Around 13,571 DMCs were placed in the gene body. The genomic distribution of CpGs on the CpG island and neighborhood context, as well as the functional context (Supplementary Figure S2a and S2b, respectively), highlighted higher methylation in cases compared to controls, mainly within the first exon, 5′UTR, and the TSS200 and TSS1500 regions (Figure 2a). After stratifying by biotype, significant differences between the ALL and control groups were observed in CpGs within lncRNAs (*p* = 2.6 × 10^−8^, d = −0.30) and protein-coding genes (*p* = 3.6 × 10^−8^, d = −0.01). In contrast, miRNAs, snRNAs, snoRNAs, and pseudogene biotypes showed no statistically significant differences (Figure 2b). The non-coding genes included 277 long non-coding RNA (*CCDC26*, *PVT1*, *CASC15*, *LINC00426*, *MEG3*, etc.), 133 microRNAs (*MIR124-2*, *MIR3134*, *MIR5095*, *MIR548F3*, *MIR548H4*, etc.), and 96 pseudogenes (*SEC1P*, *RNU6-79P*, *FMO6P*, *HLA-DPB2*, *PLEKHA8P1*, etc.) (Supplementary Table S2).

Sixteen probes of the top twenty DMCs that displayed significant differences between ALL cases and controls were located in the bodies of and *SLC2A9*, *SLC25A2*, *TK1*, *SBF2*, *PTPN14*, *CHST1*, *MBD1*, *SLC25A2*, *APP*, *OR5A2*, *SALL3*, *SOX30*, *AOAH*, *GALNT2*, *AURKC*, and *SLC26A9* genes (Table 2). Based on the number of DMCs, *MAD1L1* (*n* = 72), *RPTOR* (*n* = 55), *PCDHGA4* (*n* = 45), *TBCD* (*n* = 40), *PTPRN2* (*n* = 38), and *ZIC4* (*n* = 32) showed the highest numbers of DMCs *per gene*, in contrast to controls (Supplementary Table S2). Notably, *MAD1L1* were less methylated in patients than the ALL-free group (Supplementary Figure S3). Additionally, we found abnormal methylation patterns of commonly reported genes such as *BLK*, *BCR*, and *ARID5B*, and, notably, *MACROD2* and *LEPR* displayed highly methylated promoter regions in ALL cases (Supplementary Figure S4a–e).

#### 2.2.1. Distribution of Differentially Methylated Regions in the Functional Context

By considering that differentially methylated regions (DMRs) can affect gene expression, particularly that located in promoter regions, a primary comparison between ALL patients as cases and non-ALL subjects as controls was performed. DMRs were defined as contiguous segments containing at least seven DMCs of the genome to ensure robust regional changes [46]. This analysis revealed a total of 1056 DMR between cases and controls (mean width = 812 bp, range = 50 to 2590 pb, *p* = 0 to 0.02). The majority of them (1026: 97.2%) were hypermethylated (mean Δβ = −2.9 *p* = 0.001), while only 31 (2.8%) were hypomethylated (mean Δβ = 2.75 and *p* = 0.002) regions (Supplementary Table S3). Notably, chromosomal distribution analysis revealed that chromosome 6 harbored the highest number of DMRs (*n* = 48), while no DMRs were identified on chromosome 9.

#### 2.2.2. Pathway Enrichment and Protein–Protein Interaction Network Analysis

Pathway enrichment analysis using Gene Ontology (GO) showed that differentially methylated genes are involved in the biological processes associated with cancer, such as cell cycle regulation, apoptosis, cell proliferation, and cytokine or growth factor signaling.

**Figure 3:**
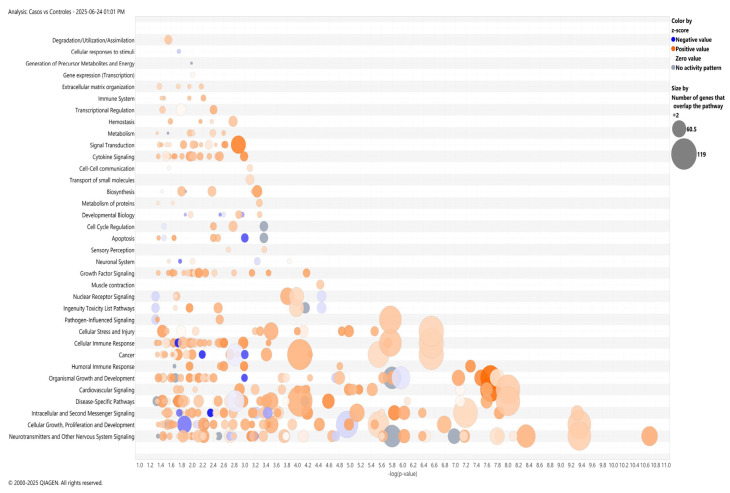
Gene set enrichment analysis on the genes showing differentially methylated CpGs between ALL patients and controls using ingenuity pathway analysis (IPA). Plot shows the most significant terms for cellular components, biological processes, and molecular functions from the IPAs of DMC-annotated genes (*p*-values  <  0.05). X-axis: Statistical significance (−log_10_(*p*)), where higher values indicate greater relevance. Bubble size represents the number of genes involved in each pathway. Color indicates the IPA z-score, which predicts the direction of regulation. Orange: significant activation (z-score > 2). Blue: significant inhibition (z-score < −2). Gray: statistically significant pathways (*p* < 0.05) with no predicted direction of change.

In addition to this, the ingenuity pathway analysis (IPA) included cancer-associated canonical pathways, cellular stress and injury, cellular immune response, and humoral immunity (Figure 3). Notably, neurotransmission and neuronal signaling emerged as the top canonical pathways, with specific enrichment in GABAergic (z-score = −0.831), serotonergic (z-score = 2.303), and glutamatergic pathways (z-score = 2.168) (65 metabolism-related genes). Additionally, IPA predicts the activation of cancer drug resistance by drug efflux (z-score = +2.353).

Additionally, IPA network analysis highlighted the involvement of inflammatory cytokines (e.g., *TNF*, *IL15*) and transcription factors (e.g., SP1) as potential modulators of cytoskeletal organization, cell chemotaxis, and activation of blood cells’ biological processes (Supplementary Figure S5).

To obtain further insight into the interaction among differential methylated genes, protein–protein interaction (PPI) network construction and identification of hub genes were performed using the STRING database. The PPI network consisted of 1287 nodes interacting at 374 edges (evidence, high confidence 0.9). The top four hub nodes with higher degrees were *GNAS* (*n* = 25), *EGFR* (*n* = 14), *SST* (*n* = 11), and *ADCYAP1* (*n* = 10), which were considered the hub proteins (Supplementary Figure S6).

### 2.3. Methylation Analysis Based on Clinical Variables

Stratification analysis based on clinical variables showed no statistically significant differences after gender, white blood cell (WBC), NCI risk, body mass index (BMI), and outcome (relapse + death) (Supplementary Table S4). Nevertheless, a comparative analysis between *ETV6*::*RUNX1*+ vs. no-*ETV6*::*RUNX1*, as well as *TCF3::PBX1*+ vs. no-*TCF3*::PBX1, revealed 4616 and 1954 DMCs, respectively (Supplementary Tables S5 and S6).

*PER2*, *STK24*, *ERI3*, *FAM19A5*, *NARFL*, *CUX1*, *TBC1D10A*, *MRPS27*, and *CBFA3T3*, were among the most hypomethylated genes in *ETV6*::*RUNX1*, while *IL10RA* and *PTGER4* were significantly hypermethylated (Supplementary Figure S7). Based on β values, the top 100 DMCs clearly distinguish *ETV6*::*RUNX1*-positive from -negative cases (Supplementary Figure S8). The GO top enriched terms identified were modulation of chemical synaptic transmission, regulation of trans-synaptic signaling, cell fate commitment, and cell junction assembly, among others (Supplementary Figure S9).

Regarding *TCF3*::*PBX1* molecular subtype, 100 DMCs were used to build the heatmap (Supplementary Figure S10). DMCs characterizing the *TCF3*::*PBX1*-positive group were located in the hypomethylated genes *DTX4*, *SLCO4C1*, *TMEM131*, *STM1*, *LINC00939*, *DHTKD1*, and *UBAC2*, and the hypermethylated genes *XXYLT1* and *PDE3B* (Supplementary Figure S11). Enrichment analysis revealed that genes with altered methylation patterns in *TCF3*::*PBX1* play a role in the regulation of neuron projection development, regulation of GTPase activity, and small GTPase-mediated signal transduction, among other processes (Supplementary Figure S12).

### 2.4. Differentially Methylated CpGs Associated with Relapse

**Figure 4:**
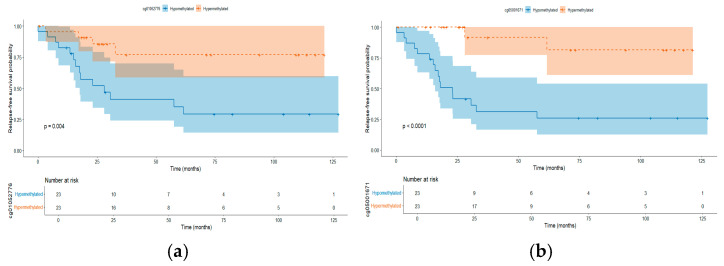

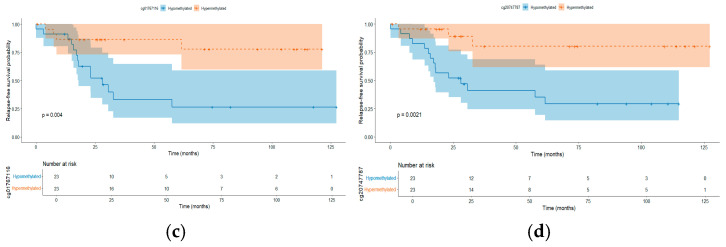
Kaplan–Meier survival curves of the most differentially methylated CpGs between ALL detected in the comparative analysis between patients and controls. Patients were categorized into low- and high-methylation groups using the median values of (**a**) cg01052776, (**b**) cg01767116, (**c**) 271 cg05001671, and (**d**) cg20747787. *p*-value was calculated using the Cox regression model, and HR indicates hazard ratio. Patients with hypermethylated levels (red lines) in any of these CpGs showed a significantly reduced risk of relapse compared to those with hypomethylated (blue lines).

To identify relapse-associated methylation patterns or CpGs at diagnosis, only cases who were followed-up at least 18 months after the initial treatment and had complete medical records were included, accounting for 28 non-relapse patients and 18 relapsed cases, who were grouped into control and case groups, respectively. Comparative analysis showed 360 CpGs (*p* < 0.05); nevertheless, none of them remain statistically significant after a multiple-correction test (FDR > 0.05). Because the CpGs cg01052776 (*p* = 0.3), cg20747787 (*p* = 0.3), cg05001671 (*p* = 0.3), and cg01767116 (*p* = 0.5) showed a trend towards differential methylation between both groups, an association analysis between their methylation levels and relapse-free survival (RFS) using univariate Cox proportional hazards regression was conducted. This approach let us use methylation as a continuous variable, and the relationship between CpGs methylation with the time-to-event outcome was directly tested. Based on this analysis, an association between all these CpGs and the risk of relapse was observed: Hazard ratio (HR) = 0.53 (95% CI: 0.37–0.75, *p* =0.0004), 0.46 (95% CI: 0.31–0.67, *p* = 0.00005), 0.50 (95% CI: 0.34–0.72, *p* = 0.0001), and 0.53 (95% CI: 0.36–0.77, *p* = 0.0008) for cg01052776, cg20747787, cg05001671, and cg01767116, respectively. Further, we examined whether CpG sites remained associated with relapse risk after adjustment for clinical variables (age, sex, white blood cell count, and NCI risk group). The adjusted hazard ratios (HRs) ranged from 0.37 to 0.45 (*p* < 0.01), indicating that each standard deviation increase in methylation was associated with a 55–63% reduction in relapse risk, independent of clinical factors (Supplementary Table S7). While these analyses share the statistical power limitations of our cohort size, the consistent and robust associations strengthen the evidence for these CpGs as candidates for future validation. Additionally, we dichotomized the methylation levels of the four CpGs, using the median value of each one to classify patients into hypomethylated and hypermethylated groups. Hypomethylation of these CpG sites was associated with worse prognosis (Figure 4a–d).

#### 2.4.1. Methylated CpGs Profile in Early Relapse

**Figure 5:**
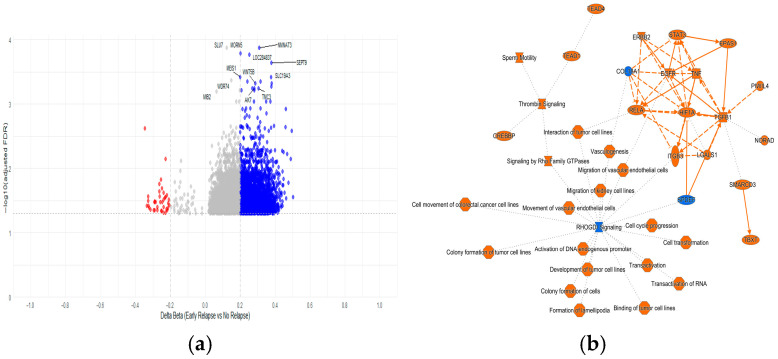
Methylation comparative analysis between ALL cases with early relapse (ER) (*n* = 7) and without relapse (*n* = 28) showing the hypomethylation and activation of Rho GTPase signaling in the ER group. (**a**) Volcano plot displaying differentially methylated CpGs (*N* = 2629) between ER and no-relapse showing statistical significance (*p* < 0.05). The x-axis shows the methylation difference (Δβ), and the y-axis represents –log10 of the adjusted *p*-value. Hypermethylated (red) and hypomethylated (blue) CpGs are highlighted. Gray points represent the non-significant DMP. (**b**) Summary graph generated by IPA comparing ER and non-relapse ALL cases. Orange color predicts activation, and blue color indicates inhibition. Octagons: biological functions; ovals: transcription regulators; squares: cytokines or growth factors; diamonds: G-protein-coupled receptors; triangles: transcription regulators with unknown or indirect roles. Solid lines: direct relationships; dashed lines: indirect relationships; dotted lines: predicted or less certain associations.

The analysis between ER and non-relapsed patients led us to identify 2629 DMCs (38 hypermethylated and 2591 hypomethylated) at FDR < 0.05. The top hypermethylated genes included *ASB18*, *SLC38A11 FAM19A2*, *TAL1*, *LOC389458*, *FAM135B*, *BCL11B*, *NR4A2*, *SLC15A1*, and *MOSC2*, while the top hypomethylated genes were *NMNAT3*, *MORN5*, *LOC284837*, *SEPT9*, *MEIS1*, *WNT5B*, *SLC19A3*, *AK7*, and *TMC3* (Figure 5a, Supplementary Table S8). The genes with more DMCs were *CORO1B* (*n* = 7), *DOCK1* (*n* = 7), *ARID5B* (*n* = 7), *MAD1L1* (*n* = 6), and *SEPT9* (*n* = 5). In addition to the genes involved in methylation processes, *UHRF1* and *DNMT3A* were found to be differentially methylated genes between the relapse and non-relapse groups. Both genes showed two DMCs located into the gene body and 5′UTR hypomethylated in the early-relapse group.

Pathway enrichment analysis showed the RhoGDI-signaling as the main abnormally methylated pathway (Supplementary Figure S13). Among the most significant canonical pathways were hepatic stellate cell activation, epithelial adherens junction signaling, Rho GTPase cycle, Sertoli cell–Sertoli cell junction signaling, and regulation of actin based motility by Rho. The most enriched canonical pathways were associated with organismal growth and development, intracellular and second messenger signaling, extracellular matrix organization, signal transduction, nuclear receptor signaling, and cancer. Notably, pathways related to neurotransmitter signaling and nervous system function ranked among the top hits (Figure 5b).

#### 2.4.2. Random Forest Model on Risk of Relapse

The 20 most significant DMCs were combined with clinically relevant variables and used to construct a Random Forest model in order to predict relapse. The highest contribution observed included four CpGs (cg01052776, cg20747787, cg05001671, and cg01767116), which displayed MeanDecreaseAccuracy values from 12 to 14. The model achieved a training accuracy of 97.5% (95% CI: 94.1–99.9) and an out-of-bag (OOB) error rate of 5% (95% CI: 2.1–9.8). Given the limited sample size (18/46 relapse events), we relied solely on OOB error estimation for internal validation, as holdout validation would have further reduced the already limited training data. The model achieved an OOB accuracy of 78.3%, with a sensitivity of 90.0% for detecting relapse cases and a specificity of 75% for non-relapse cases. The confusion matrix showed that nine out of ten relapse cases were correctly identified, but nine out of thirty-six non-relapse cases were misclassified as relapse (false positives). Variable importance analysis confirmed that the four CpG sites (cg01052776, cg20747787, cg05001671, cg01767116) were among the top predictors. A near-perfect separation of outcome groups and reduced performance in the holdout validation set (75% accuracy) were signs of overfitting; therefore, the model requires further validation in larger cohorts for biomarker discovery and relapse prediction.

## 3. Discussion

Hematological malignancies, including ALL, are multifactorial entities in which epigenetic factors such as DNAme play a significant role [24,47,48]. Despite the enormous advances in the success of ALL treatments, this disease remains the leading cause of cancer-related death in children and adolescents, with the Mexican pediatric population being among the most affected worldwide [1,3,4,6,18]. In fact, Mexico reports one of the highest incidence and mortality rates of ALL [3,6,18]. A more developed knowledge of the genetic and epigenetic processes undergoing leukemogenesis and therapy responsiveness is a cornerstone of the new treatment identification of this disease. To obtain a better understanding of ALL Mexican patients, we performed a case–control study involving pediatric cases under 17 years old. Based on the fact that PB samples could provide similar information to BM in leukemia [49] and also based on the low number of DMCs (<0.01%) detected between both tissues, we included samples from PB or BM for further analysis. Our study identifies more hypermethylated than hypomethylated DMRs in children with ALL, in contrast to controls (Supplementary Table S3). Genome-wide abnormalities in DNAme are a hallmark of cancer [24,47,50], and increased DNAme in normally unmethylated genes has been already reported in ALL [50,51,52,53,54,55]. It has been described that global hypomethylation, observed in most types of cancer, could be associated with the loss of methylation during each cell division as a defense mechanism of the cell in the course of tumor progression [51,56]. The high hypermethylation rate in ALL could be explained by the rapid evolution progress of this disease, which occurs within weeks, and as a result of a failure of leukemic cells to de-activate tumor-suppressor mechanisms [51,57,58].

Genes that have been reported as abnormally methylated in leukemia, including *CPEB1*, *BLK*, *FLT3*, *PAX5*, *EBF1*, *HDACs*, *IKZF1*, *RB*, etc., were also detected in this study [41,48,50,59,60,61,62,63]. These genes are relevant for hematopoietic cell commitment, and abnormal expression of them have been associated with ALL outcome [64,65,66].

As far as we known, few EWAS using the MethylationEPIC Beadchip genome-wide DNAme arrays (850K) in ALL have been conducted [39], and several of them have used the 4500K beadchip. In addition to the consistency of well-known abnormally methylated genes in leukemia cells, these studies uncovered new likely essential genes for leukemogenesis. As an example, *VTRNA2-1* was found to be hypermethylated at birth and at diagnosis in ALL cases, and, interestingly, normal levels were found at remission, but increased levels were found at relapse [34].

Another EWAS performed in drops of blood collected at birth in children with Down Syndrome (DS) showed that *HOPX*, *SMIM24*, and *PPP1R10* were abnormally methylated in children with DS who developed ALL [39].

In the present study, we identify *MAD1L1*, *RPTOR*, *PCDHGA4*, *TBCD*, and *PTPRN2* as the genes with the highest count of DMCs between cases and controls, while *PTPN14* and *SLC2A1* were identified as having the most significant DMCs.

The encoded protein *PTPRN2* is a tumor-suppressor gene involved in malignant cell transformation [67,68,69]. To our knowledge, there is no available data on the abnormal methylation of *PTPN2* in ALL, nor on other hematological diseases, even though there is no doubt as to its role in T-ALL [70]. Moreover, *PTPRN2* plays a role in vesicle-mediated secretory processes, and has been associated with progression [71] and as a methylation biomarker of poor survival in colorectal cancer [72]. Meanwhile, *RPTOR*, a crucial component of the mTOR pathway, regulates cell growth in response to nutrient and insulin levels and has been reported as a key driver gene in the brain metastasis of lung and colorectal cancer progression [72,73,74].

Another gene was the mitotic-arrest-deficient 1-like 1 protein gene, a key component of the spindle assembly checkpoint that is essential for correct chromosomal segregation during mitosis. *MAD1L1* was strongly associated with cancer progression and the development of colorectal cancer, and its aberrant expression has been reported in solid tumors [75]. In murine models, overexpression of *MAD1L1* decreases p53 levels and promotes inflammation-driven colon tumorigenesis [76], while altered expression of this gene has been associated with poor prognosis and recurrence in hepatocellular carcinoma [77]. However, methylation data in ALL or any hematological disease have been scarcely explored. Notably, abnormal methylation at *MAD1L1* (loci cg08972190) has been reported in children surviving ALL, particularly in association with obesity and exposure to cranial radiotherapy [78]. In addition, DMRs of *MAD1L1* have also been published in adults with ALL [79]. In addition to these findings, in our data, *MAD1L1* emerges as a relevant candidate gene in leukemogenesis. Based on our results and data from the analysis of *PTPN14* in diverse types of cancer, we suggest that epigenetic mechanisms could modulate gene expression [43,48]; we also propose that aberrant methylation of this gene could contribute to leukemogenesis.

Similarly to most cancer types, aberrant methylation patterns in the promoter regions were observed in ALL (Figure 2a) [80]. Among the genes that were most differentially hypomethylated in their promoter region are *PPP1R18*, *GNG7*, *ABR*, *PDE9A*, and *NT5C*, whose deregulation could favor the uncontrolled proliferation of blasts [81,82]. Notably, abnormal expression of *NT5C*, which is related to pyrimidine metabolism, has been associated with resistance to chemotherapies (e.g., cytarabine) in pediatric leukemia [83].

Otherwise, *MACROD2* and *LEPR* were two genes displaying highly methylated promoter regions in ALL cases. *MACROD2* is an epigenetic regulator that encodes an enzyme that removes ADP-ribose groups from mono-ADP-ribosylated proteins and deacetylates metabolites derived from histone deacetylation, such as O-acetyl-ADP ribose, influencing chromatin regulation and gene expression [84]. Meanwhile, the *LEPR* gene is associated with the JAK-STAT pathway. Interestingly, human xenograft models revealed that *LEPR* expression is essential for the development and maintenance of ALL and different types of cancer [85].

The pathway enrichment analysis confirmed the expected involvement of cancer processes such as cell cycle regulation, apoptosis, cell proliferation, and cytokine- and growth-factor-mediated signaling were the most affected in ALL [80]. Interestingly, we also detected enrichment for pathways related to neurotransmission and neuronal signaling. The biological significance of neuronal signaling in ALL is currently unknown. It is hypothesized that cancer cells, including leukemia, hijack neural pathways and can exploit neuronal networks to invade the central nervous system (CNS) [86]. Previous studies have reported methylation changes in neuropeptides in ALL [87] and in a significant proportion of patients presenting with CNS infiltration [87,88]. Additionally, the evidence of overexpression of the GABAergic pathway [89] and the usage of the serotonin and glutamatergic pathway for proliferation and survival in cell lines [90,91] support the hypothesis that the neurotransmission and neuronal signaling pathways are involved in ALL. It is noteworthy that many genes within these pathways (e.g., *AKT2*, *AKT3*, *CREBBP*, *MAPK1*, *GNAS*, *RPTOR*) are already known to play relevant roles in cancer cell growth and proliferation [74,92,93,94,95], which could provide a plausible biological basis for such a link. Notwithstanding, functional studies are required to decipher any potential causal relationship.

In addition, we investigated whether methylation patterns might be associated with *ETV6*::*RUNX1* and *TCF3*::*PBX1* molecular subtypes or relapse risk. Notably, 3 out of the 42 CpGs previously proposed by Nordlund to define *ETV6*::*RUNX1* and 16 out of the 21 CpGs proposed for *TCF3*::*PBX1* were also found in our analysis, highlighting the existence of specific patterns of DNAme based on fusion genes, potentially affecting prognosis [40].

The analysis of outcomes revealed four CpGs associated with RFS: two of them were found in the coding genes *RNH1* and *FBXL22*. Although associations between DNAme and relapse have been proposed in pediatric ALL, few studies have directly assessed DMCs between patients with and without relapse. One of the most relevant contributions [40] employed a shrunken centroid classification followed by RFS analysis, identifying gene regions with methylation levels associated with RFS. *RNH1* encodes a ribonuclease inhibitor that has been reported as being aberrantly expressed in bladder cancer [96]. Interestingly, this has also been suggested as a therapeutic target due to its role in mTOR signaling [97]. *FBXL22* encodes an F-box protein that interacts with S-phase kinase-associated protein 1A and cullin to form SCF (Skp1-Cullin-F-box) complexes functioning as ubiquitin ligases. This gene has been linked to breast cancer [98].

The consistent performance of the four candidate CpG sites in multivariate analyses represents a particularly noteworthy finding. Despite the limited sample size for multivariable modeling, all four CpGs maintained significant associations with RFS after adjustment for established clinical risk factors including age, white blood cell count, and NCI risk category. To the best of our knowledge, the present study is among the first describing the DNAme landscape of relapsed cases at diagnosis. Nordlund [99] included 764 ALL cases to explore the association between DNAme with RFS based on the most common molecular subtypes; six, eight, and one regions at FDR < 0.1 were found in *ETV6*::*RUNX1*, *KMT2A*-r and *BCR*::*ABL1*, respectively [99]. Other studies have compared paired diagnosis-first relapsed samples, finding significant differences between them and suggesting that DNAme can propel leukemia evolution [100,101]. Otherwise, we were unable to develop a multivariate approach for relapse prediction, as Mosquera-Orgeira did (relapse risk prediction index composed by16 CpGs, 20-month AUC with 81.4% and 82.5%) [102]. In our Random Forest analysis, the near-perfect separation observed in the training set and the drop in performance in the test set clearly indicate model overfitting [103,104]. This is likely attributable to (i) the limited sample size, (ii) the high dimensionality of the methylation dataset, and (iii) class imbalance [103,104]. Nonetheless, the consistent identification of four relapse-associated CpGs within this Cox univariated and multivariate model warrants further evaluation on their prognostic relevance. These findings require validation in larger independent cohorts before any predictive or clinical application can be considered.

After the analysis stratification by relapse type, *DNMT3A* and *UHRF1*, which are a DNAme writer and modulator, respectively, were identified in ER. *UHRF1* encodes a member of a subfamily of RING-finger type E3 ubiquitin ligases that acts as a key epigenetic regulator by bridging DNAme and chromatin modification. In the present study, we observed significant hypermethylation at 14 CpGs within the gene body of *UHRF1* in ALL patients with ER compared to non-relapsing patients. This finding suggests a potential disruption in the epigenetic landscape during ER, positioning *UHRF1* as a candidate biomarker for risk to ER [105]. To note, *UHRF1* has been identified as a hub gene in chronic myeloid leukemia (upregulated in murine models) [106], and has been reported as a promoter of oncogenesis in T-cell ALL. Further, *UHRF1* drives proliferation in T-cell ALL, and has been proposed as a therapeutic target for thymoquinone, a natural anticancer agent [107]. While *UHRF1* methylation changes could lead to increased gene expression, altered mRNA splicing, or aberrant protein isoforms, functional studies are needed for unveiling the mechanism underlying ER in ALL [107].

In summary, our study provides a comprehensive epigenomic overview of pediatric ALL in the Mexican population, revealing aberrant methylation patterns associated with leukemogenesis, molecular subtypes, and ER risk. The identification of potential biomarkers with relapse and novel candidate genes, including *UHRF1* and *MAD1L1*’s involvement in ER, underscores the contribution of epigenetic dysregulation to disease progression and relapse dynamics. Although relapse prediction using DMCs alone proved to be limited in our cohort, stratified analyzes suggest that subtype-specific methylation signatures may hold prognostic value. We are aware that the small sample size included in the present study is a critical limitation, which could be amplified due to tissue heterogeneity or after stratification by molecular subtype analyses (e.g., relapse, fusion subtypes). Therefore, to confirm our results and the clinical utility of the proposed potential epigenetic biomarkers, validation in larger and independent cohorts, as well as validation matched by variables (e.g., type tissue, age, gender) that could potentially bias the results, is needed. Additionally, these findings highlight the need for integrative approaches combining epigenetic, genetic, and clinical data to improve risk stratification and therapeutic targeting in childhood ALL.

## 4. Materials and Methods

### 4.1. Patient and Sample Collection

Newly diagnosed patients with B-ALL and individuals without ALL (used as controls), aged 0–17 years, and without Down syndrome were included in this study. Bone marrow (BM) or PB samples were collected at the time of diagnosis and prior to treatment initiation. Samples under 75% of lymphoblast count in BM or PB were excluded. Controls consisted of individuals aged 0–17 years who underwent BM aspiration as part of a differential diagnostic process due to clinical suspicions of acute leukemia, but were confirmed not to have ALL. No specific age or sex matching was performed.

Public hospitals affiliated with the Mexican Inter-Institutional Group for the Identification of the Causes of Childhood Leukemia (MIGICCL) contributed patients to this study. The diagnosis of B-ALL was validated by pediatric hematologists/oncologists based on clinical presentation, and was confirmed using BM aspirate analysis, which included morphological assessment, immunophenotyping, and, when available molecular studies, in accordance with the 2022 World Health Organization (WHO) classification of lymphoid neoplasms. Cohort follow-up began on the date of B-ALL diagnosis confirmation (Day 0) and continued until the date of the last hospital visit or death.

Clinical and demographic data were obtained from the patients’ medical records. The variables analyzed included the following: patient’s sex, age at diagnosis, National Cancer Institute (NCI) risk classification (standard risk, high risk), CNS and/or testicular infiltration at diagnosis, gene rearrangements (when available), death during induction (yes/no), early mortality (yes/no), and causes of death. Gene rearrangement data were limited to four fusion genes (*ETV6::RUNX1*, *TCF3::PBX1*, *BCR::ABL1*, and *KMT2A::AFF1*), which are the only cytogenetic and molecular biomarkers tested in Mexican public hospitals.

NCI risk classification: The risk was assigned based on age and peripheral white blood cell (NCI) count at diagnosis. Patients aged between 1.00 and 9.99 years with a WBC count < 50 × 10^9^/L were classified as standard risk. In contrast, those aged ≤1 year or ≥10 years and/or with a WBC count ≥ 50 × 10^9^/L were classified as high risk, in accordance with established criteria [108].

Relapse was defined as disease recurrence in patients who had previously achieved morphologic remission, characterized by <5% blasts in BM and clearance of extramedullary disease [9]. Bone marrow relapse was defined as ≥25% morphologic blasts or ≥5% blasts accompanied by extramedullary involvement. Central nervous system relapse was identified based on CNS3 status (≥5 white blood cells/μL in cerebrospinal fluid with blasts on cytospin) or the presence of clinical signs of CNS leukemia. Isolated extramedullary recurrence required histological confirmation by biopsy. Based on the time to relapse following treatment initiation, patients were categorized into two groups: very early relapse (VER: <18 months) and early relapse (ER: 18–36 months) [9,64].

### 4.2. Nucleic Acid Extraction and Fusion Gene Analysis

Nucleic acids were extracted from PB or BM samples. DNA extraction was performed using the Puregene Blood Core Kit (QIAGEN, Hilden, Germany), according to the manufacturer’s instructions. Nucleic acid integrity was assessed by 1% agarose gel electrophoresis. The four most common fusion genes (*ETV6::RUNX1*, *TCF3::PBX1*, *BCR::ABL1*, and *KMT2A::AFF1*) were detected using methods previously described [21].

### 4.3. Genome-Wide DNA Methylation Analysis

Genome-wide DNAme profiling was performed using the Infinium MethylationEPIC v2.0 BeadChip array (Illumina, San Diego, CA, USA), which interrogates over 850,000 CpG sites across gene promoters, gene-coding and -enhancer regions, miRNA-promoter regions, and intergenic CpGs. A total of 500 ng of genomic DNA per sample was subjected to bisulfite conversion using the EZ DNA Methylation Kit (Zymo Research, Irvine, CA, USA) according to the manufacturer’s instructions. The bisulfite-converted DNA was then hybridized to the EPIC arrays. Array scanning and image acquisition were carried out using the Illumina HiScan SQ system (Illumina Inc., San Diego, CA, USA), which generated raw intensity data in the form of IDAT files. Initial quality control of the IDAT files was conducted using BeadArray Controls Reporter (Illumina, San Diego, CA, USA). Data preprocessing and normalization were performed in R (v4.3.0) using the ChAMP pipeline (Chip Analysis Methylation Pipeline, v2.23.0) for EPIC arrays, available from Bioconductor v3.18 (https://bioconductor.org/packages/release/bioc/html/ChAMP.html accessed on 19 October, 2025) [46,109,110]. Probes with detection *p*-values ≥ 0.01 in more than 5% of samples, indicating poor signal reliability, were discarded. Additionally, we removed non-CpG probes, probes containing single-nucleotide polymorphisms (SNPs) at the CpG site or within the single-base extension, multi-hit probes aligning to multiple genomic locations, and all probes located on sex chromosomes (X and Y) to avoid confounding by sex differences. Samples with more than 10% of probes failing the detection *p*-value threshold (<0.01) were excluded. Intra-array normalization was performed using the Beta MIxture Quantile dilation (BMIQ) method to correct for the well-documented type I/II probe design bias [46]. To account for potential technical variation, we applied the ComBat algorithm from the sva R package, using the pd$Sample_Plate variable as the known batch covariate. Model adjustment for known biological covariates (e.g., age, sex) was not performed, as our primary aim was to identify disease-associated signals. Methylation levels were quantified as β values, calculated as the ratio of the methylated signal intensity to the sum of methylated and unmethylated signal intensities for each CpG site. These β values ranged from 0 (completely unmethylated) to 1 (fully methylated), reflecting the proportion of DNA methylation at individual CpG loci [46]. Preprocessing steps included filtering out probes with detection *p*-values < 0.01, non-CpG probes, probes overlapping known SNPs, multi-hit probes, and probes located on sex chromosomes (X and Y). Normalization of β values was performed using the Beta MIxture Quantile dilation (BMIQ) method [46], and batch effects were corrected using the ComBat algorithm [46]. The GRCh37/hg19 human genome assembly was used as the reference for probe mapping and gene annotation.

### 4.4. Assessment of Differentially Methylated Regions

Since BM or PB samples were available, we first evaluated global DNAme differences between these two tissue types to identify potential biases and ensure that the analyzed DNAme signal was representative of the leukemic clone, minimizing confounding signals from the normal cellular background and potential differences between tissue microenvironments. A multidimensional scaling plot based on the 1000 most variable CpG sites was generated using the champ.QC function from the ChAMP package.

To identify DMCs, the champ.DMP function was applied to compare B-ALL cases vs. controls, as well as to perform stratified analyzes based on clinical features. P-values were adjusted for multiple testing using the Benjamini–Hochberg method. CpG probes with an absolute Δβ (|Δβ|) ≥0.2 and an adjusted *p*-value <0.05 were considered statistically significant. Heatmap was constructed using ComplexHeatmap (V 2.16.0), and Volcano plots were constructed using ggplot2 (V 3.5.2) in R.

DMRs were identified using the champ.DMR function within the ChAMP pipeline, which applies the bumphunter algorithm to detect the genomic regions with consistent methylation differences between groups. DMRs were defined as contiguous genomic segments meeting the following criteria: each DMR contained at least 7 DMCs, ensuring robust regional methylation changes. Adjacent DMCs were grouped into the same DMR if they were located within ≤50 bp of each other, based on the assumption that regulatory elements typically span short genomic intervals. DMRs were identified after 250 bootstrap interactions to estimate the null distribution of methylation differences [46,111].

### 4.5. Epigenome-Wide Association Study (EWAS)

Global DNAme differences were assessed between cases and controls, as well as by stratifying patients according to gender, hyperleukocytosis (WBC >100,000 vs. <100,000), NCI risk classification, BMI z-score category (normal weight, underweight, obesity), gene fusion (*ETV6*::*RUNX1* and *TFC3*::*PBX1*), and clinical outcome (relapse or death).

DNA methylation differences between relapse and non-relapse ALL patients was also assessed. Adjusted *p*-value threshold of 0.05 was considered statistically significant. Selected CpG sites were further analyzed using a univariate Cox proportional hazards regression mode to evaluate RFS. This analysis incorporated both time to relapse and relapse status (coded as follows: 1 = event occurred; 0 = censored at last follow-up). *p*-values were adjusted for multiple comparisons using the Benjamini–Hochberg false discovery rate (FDR) method. Further, these CpG sites were analyzed using Kaplan–Meier survival analysis and the Mantel–Cox test. Methylation β values were dichotomized based on the overall mean to classify samples as hypomethylated or hypermethylated.

To assess whether the association of the four candidate CpG sites with relapse was independent of the established clinical risk factors, we performed exploratory multivariate Cox proportional hazards analyses. Each CpG site was evaluated in a separate model adjusted for age at diagnosis (continuous variable), sex, white blood cell count (categorized as <50,000/µL vs. ≥50,000/µL and <100,000/µL vs. ≥100,000/µL), and NCI risk category (standard risk and high risk). Methylation levels for each CpG were included as continuous variables scaled to standard-deviation units to facilitate the interpretation of HR.

### 4.6. Relapse Prediction Model in ALL Patients, Grounded in a Random Forest-Based Machine Learning Approach

To evaluate the predictive potential of the top 20 DMCs, a Random Forest classifier was implemented following these steps: (1) Data partitioning: The dataset was split into a training set (75%) and a holdout test set (25%), with stratification by relapse status to preserve class distribution. (2) Model training: The Random Forest model was trained using 1,000 decision trees. The number of variables randomly sampled at each split (mtry) was optimized through 5-fold cross-validation. Variable importance was assessed using the Mean Decrease in Accuracy and Mean Decrease in Gini metrics. (3) Performance evaluation: Model performance was evaluated on the independent test set using area under the ROC curve (AUC-ROC), sensitivity, and specificity. To ensure the validity of the Cox proportional hazards analysis, proportionality assumptions were verified using Schoenfeld residuals. Additionally, potential model overfitting in the Random Forest classifier was mitigated using out-of-bag (OOB) error estimation and holdout test validation [112].

### 4.7. Pathway Enrichment and Protein–Protein Interaction Analyses

Pathway enrichment analysis was performed using the GO database (http://www.geneontology.org accessed on 25 July 2025) and IPA software (QIAGEN inc., https://digitalinsights.qiagen.com/IPA accessed on 25 July 2025) to identify the most significantly altered biological pathways in ALL [113,114]. GO provides comprehensive gene annotations, including molecular functions, biological processes, and cellular components, and IPA calculates enrichment scores (*p*-values) based on the probability of gene set overrepresentation. For each probe, the associated gene symbol, Δβ-value, and adjusted *p*-value were used as input. Canonical pathways were identified using enrichment *p*-values calculated by Fisher’s exact test. Genes were considered biologically relevant if their associated CpG sites showed a methylation change (Δβ) of ≥±0.20 and were located in functionally relevant genomic regions (e.g., promoter regions, CpG islands).

To evaluate protein–protein interaction (PPI) networks among genes harboring DMCs in ALL versus controls, we used the STRING database (https://string-db.org, accessed on 29 June 2025). Hub genes were identified, and high-confidence interaction networks were constructed using a confidence score threshold of 0.900 [115].

### 4.8. Statistical Analysis

All statistical analyzes were performed using R software version 4.3.0. Qualitative variables were expressed as counts and percentages. Comparisons between groups were made using Student’s *t*-test for continuous variables and Chi-square or Fisher’s exact test for categorical variables, as appropriate. Spearman’s rank correlation coefficient was used to assess associations between continuous or ordinal variables. To estimate effect size, Cohen’s d was calculated, which represents the standardized difference between the two group means, divided by their pooled standard deviation, and indicates the magnitude of the observed difference [116,117]. To evaluate the prognostic impact of DMCs on relapse, a univariate Cox regression analysis was conducted. For this analysis, based on the median methylation values, samples were grouped into hypomethylated or hypermethylated categories. Survival differences between these both groups were evaluated using the Mantel–Cox log rank test. Two-sided *p*-values < 0.05 were considered statistically significant.

## 5. Conclusions

This study is one of the few case–control analyses providing a comprehensive examination of DNAme patterns in pediatric patients with ALL, revealing a distinctive epigenetic signature associated with ALL. It is characterized by methylation abnormalities in genes involved in the cell cycle and cell signaling, as well as unexpected pathways such as neurotransmission. The genes involved in the latter pathway could hypothetically influence leukemic cell trafficking into the CNS and could be associated with chemotherapy drugs evasion. Even the limited statistical power of this study, due to its small sample size and chemotherapy drugs evasion, the identification of DNAme profiles associated with *ETV6*::*RUNX1* and *TCF3*::*PBX1*, two of the most common molecular subtypes of ALL, provide valuable information that could contribute to the classification of molecular subtypes in pediatric ALL. We also identified four specific CpG sites (cg01052776, cg20747787, cg05001671, and cg01767116) whose methylation levels were significantly associated with RFS, suggesting their potential use as prognostic biomarkers; however, further validation in independent cohorts is needed. Finally, our findings provide knowledge about the relevance of epigenetic mechanisms in pediatric ALL and open new opportunities for the identification of prognostic biomarkers as well as for the development of targeted therapeutic strategies.

## Figures and Tables

**Table 1:** Clinical characteristics of pediatric ALL patients.

Variable	*n* (%)
Sex	
Male	24 (45)
Female	29 (55)
Age	
1–9 years	46 (87)
>10 years	7 (13)
WBC count at diagnosis (1 × 10^3^/µL)	
<50	32 (60)
>50	21 (40)
WHO ALL classification	
*ETV6::RUNX1*	11 (20)
*TCF3::PBX1*	6 (12)
*BCR::ABL1*	2 (4)
*KMT2A::AFF1*	1 (2)
Undetermined *	33 (62)
NCI risk classification at diagnosis	
Standard risk	28 (53)
High risk	25 (47)
WHO Z-BMI	
Underweight	7 (13)
Normal weight	25 (48)
Overweight	2 (4)
Obesity	11 (20)
No data	8 (15)
Death	
Yes	8 (18)
No	38 (82)
Relapse status	
Relapse **	18(34)
VER	11 (61%)
ER	7 (39%)
Non-relapse	28 (53)
Unknown	7 (13)

WBC: white blood cell; WHO: World Health Organization; * Undetermined: 26 (49%) cases were negative to *ETV6*::*RUNX1*, *TCF3*::*PBX1*, *BCR*::*ABL1*, and *KMT2A*::*AFF1*, and the remaining 7 (13%) were not tested for the most common fusion genes; Z-BMI: Body Mass Index Z score; NCI: National Cancer Institute; ** Relapse data was available for 46/53 patients; VER: very early relapse; ER: early relapse.

**Table 2:** Top 20 DMCs defining acute lymphoblastic leukemia.

CpG ID	Chromosome	Gene	Genomic Region	CpG Island Region	Δβ	FDR
cg10030658	6		IGR	island	−0.21	7.30 × 10^−14^
cg26342454	4	*SLC2A9*	5′UTR	opensea	0.34	4.59 × 10^−13^
cg00030420	5	*SLC25A2*	TSS200	island	−0.26	2.81 × 10^−11^
cg06098276	17	*TK1*	Body	opensea	−0.25	2.81 × 10^−11^
cg07007382	6		IGR	opensea	0.26	4.24 × 10^−11^
cg03990261	11	*SBF2*	Body	opensea	−0.39	1.20 × 10^−9^
cg09974432	1	*PTPN14*	Body	opensea	0.24	1.52 × 10^−9^
cg09039751	11	*CHST1*	Body	island	−0.41	1.88 × 10^−9^
cg11093980	18	*MBD1*	Body	shore	−0.23	1.88 × 10^−9^
cg21636683	5	*SLC25A2*	TSS200	island	−0.20	2.09 × 10^−9^
cg23082883	7		IGR	opensea	−0.31	3.70 × 10^−9^
cg19423170	21	*APP*	Body	opensea	−0.32	3.70 × 10^−9^
cg00706994	11	*OR5A2*	1stExon	opensea	0.43	3.70 × 10^−9^
cg16284437	18	*SALL3*	Body	shore	−0.23	4.42 × 10^−9^
cg12919873	21		IGR	opensea	0.45	4.98 × 10^−9^
cg02918872	5	*SOX30*	TSS200	island	−0.21	6.97 × 10^−9^
cg26767916	7	*AOAH*	Body	opensea	−0.30	6.97 × 10^−9^
cg09764435	1	*GALNT2*	Body	opensea	0.34	6.97 × 10^−9^
cg25432232	19	*AURKC*	5′UTR	island	−0.31	7.01 × 10^−9^
cg16732663	1	*SLC26A9*	Body	opensea	0.50	7.36 × 10^−9^

IGR: Intergenomic region; 5′UTR: 5‘untranslated region; TSS1500/200: 1500/200 base pairs upstream of the transcription start site; 1stExon: first exon.

## Data Availability

The raw sequencing data from this study have been submitted to the NCBI Gene Expression Omnibus (GEO) database and are currently under accession number GSE306095. The dataset will be publicly accessible upon publication of this manuscript.

## Supplementary Materials

The following supporting information can be downloaded at: https://www.mdpi.com/article/10.3390/ijms262110261/s1.

## Abbreviations

The following abbreviations are used in this manuscript:

ALL	Acute lymphoblastic leukemia
DNAme	DNA methylation
EWAS	epigenome-wide association study
B-ALL	B-cell ALL
Z-BMI	Body Mass Index Z score
NCI	National Cancer Institute
VER	Very early relapse
ER	Early relapse
DMC	Differentially methylated CpG sites
5’UTR	5′ untranslated regions
TSS200	Region 200bp previous of transcription start site
TSS1500	Region 1500bp previous of transcription start site
DMR	Differentially methylated regions
GO	Gene Ontology
IPA	Ingenuity pathway analysis
PPI	Protein–protein interaction
OOB	Out-of-bag
RFS	Relapse-free survival
MIGICCL	Mexican Inter-Institutional Group for the Identification of the Causes of Childhood Leukemia
BM	Bone marrow
PB	Peripheral blood
CNS	Central nervous system
BMIQ	Beta Mixture Quantile dilation
HR	Hazard ratios
