# Supplementary material for: Comprehensive Epigenome-Wide Profiling Reveals Distinctive DNA Methylation Signatures and Potential Prognostic Biomarkers in Mexican Pediatric B-ALL
Source: Int J Mol Sci. 2025 Oct 22;26(21):10261. doi: 10.3390/ijms262110261 (PMC12607430; doi:10.3390/ijms262110261)
Supplement: Supplementary file 1 [file ijms-26-10261-s001.zip › Supplementary Figures S1—S13.pdf]

Supplementary Figures

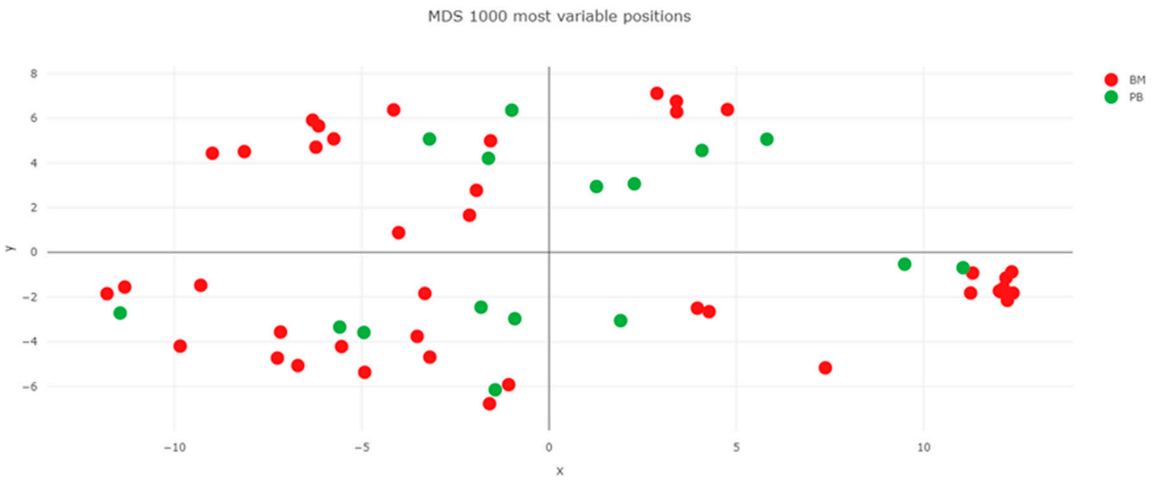

Supplementary Figure S1: Multidimensional scaling plot between peripheral blood (PB) and bone marrow (BM). Each point represents one sample, coded by group (BM in red, PB in green). The axes have no intrinsic meaning, as they represent a projection of the distances between samples into a lower dimensional space to facilitate visualization of the relationships between them.

a)

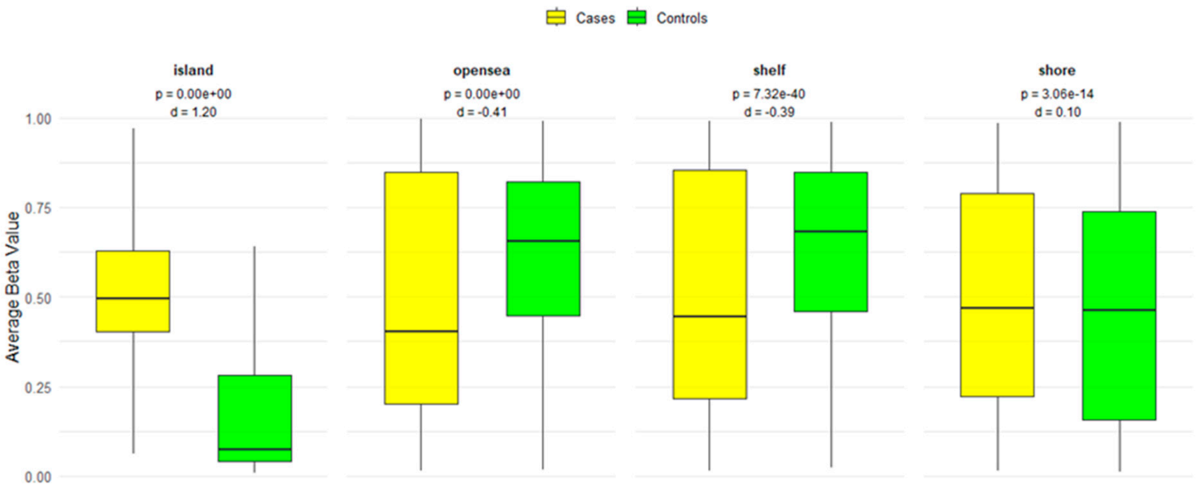

b)

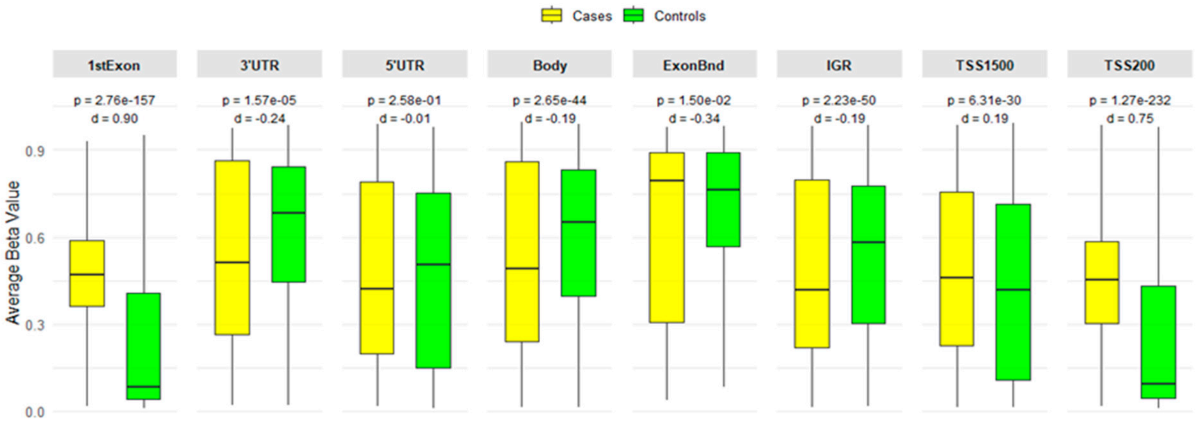

Supplementary Figure S2. Distribution of DNA methylation beta values in ALL cases and controls. a) Distribution by CpG context. b) Distribution by genomic region. P-values were obtained using the Wilcoxon test, and effect sizes were calculated using Cohen's D. Yellow boxes represent ALL cases, and green boxes represent controls. Abbreviations: 3'UTR – 3' untranslated region; 5'UTR – 5' untranslated region; TSS – transcriptional start site; IGR – intergenic region. CpG Island annotations: Island – CpG-rich regions; Shore – 0–2 kb flanking islands; Shelf – 2–4 kb flanking islands; Open Sea – distant regions with low CpG density.

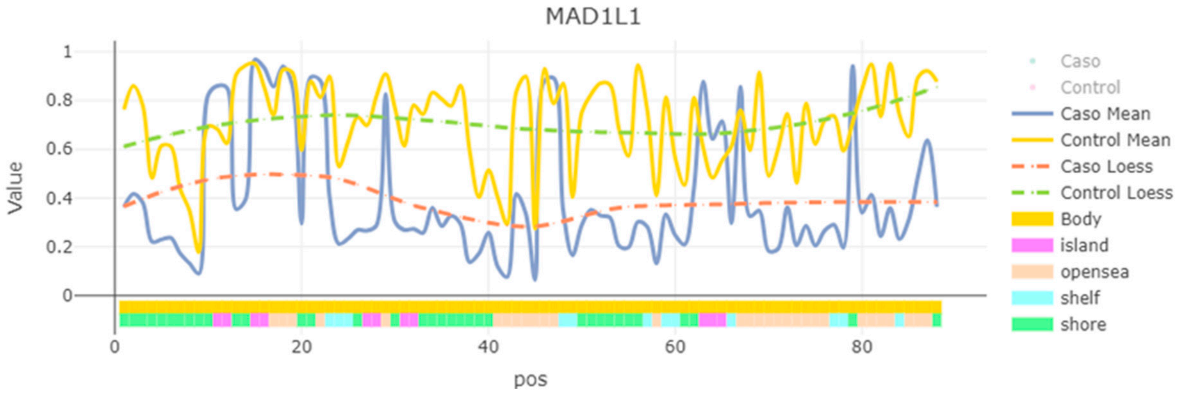

Supplementary Figure S3. DNA methylation profile of the *MAD1L1* gene. Methylation beta values of CpG sites within the *MAD1L1* gene are shown for ALL cases (yellow) and controls (blue). Solid lines represent individual samples, while dashed lines indicate less smoothed curves for each group. The lower track denotes genomic context annotations: gene body (pink), CpG island (purple), open sea (salmon), shelf (light blue), and shore (green). Genomic positions of the CpG probes are represented along the x-axis.

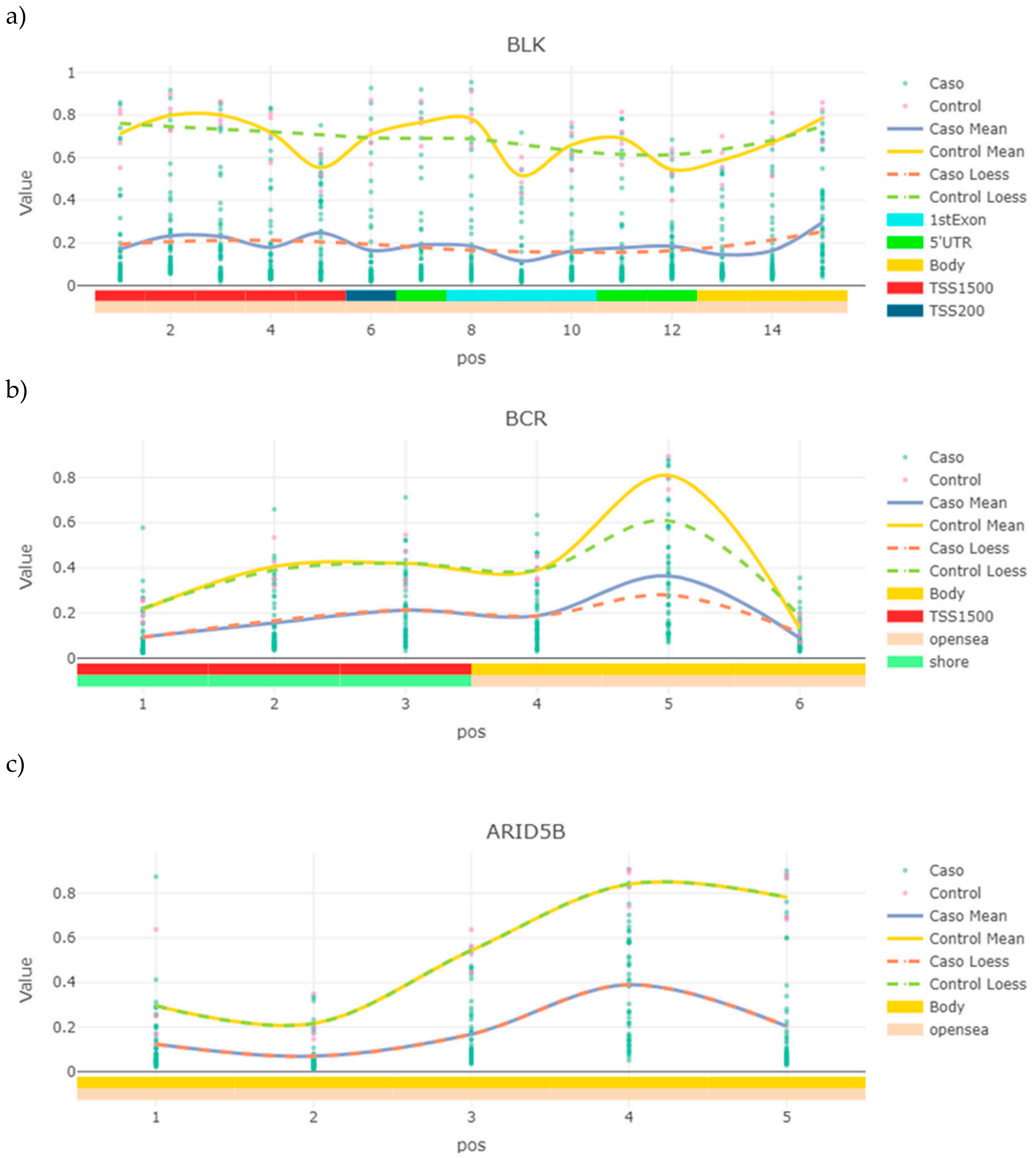
a)
BLK
Value
pos
Caso
Control
Caso Mean
Control Mean
Caso Loess
Control Loess
1stExon
5'UTR
Body
TSS1500
TSS200
b)
BCR
Value
pos
Caso
Control
Caso Mean
Control Mean
Caso Loess
Control Loess
Body
TSS1500
opensea
shore
c)
ARID5B
Value
pos
Caso
Control
Caso Mean
Control Mean
Caso Loess
Control Loess
Body
opensea

d)

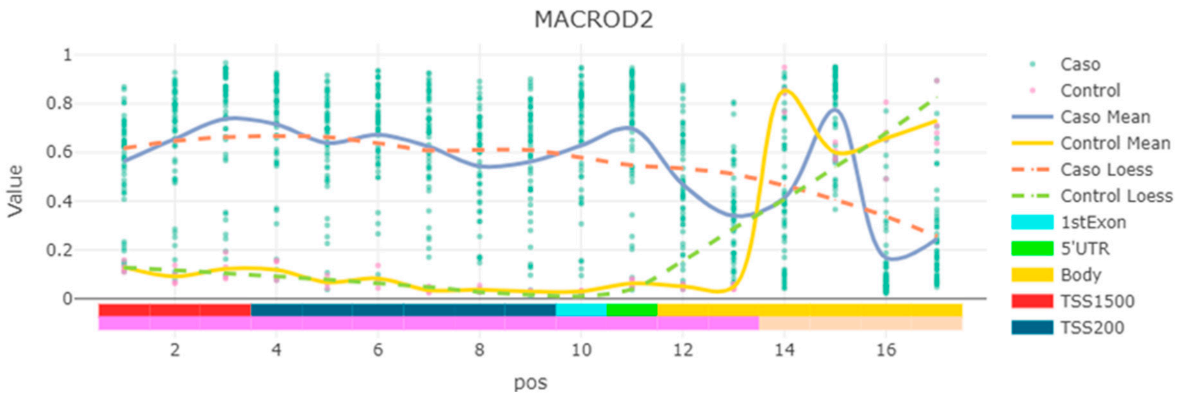

e)

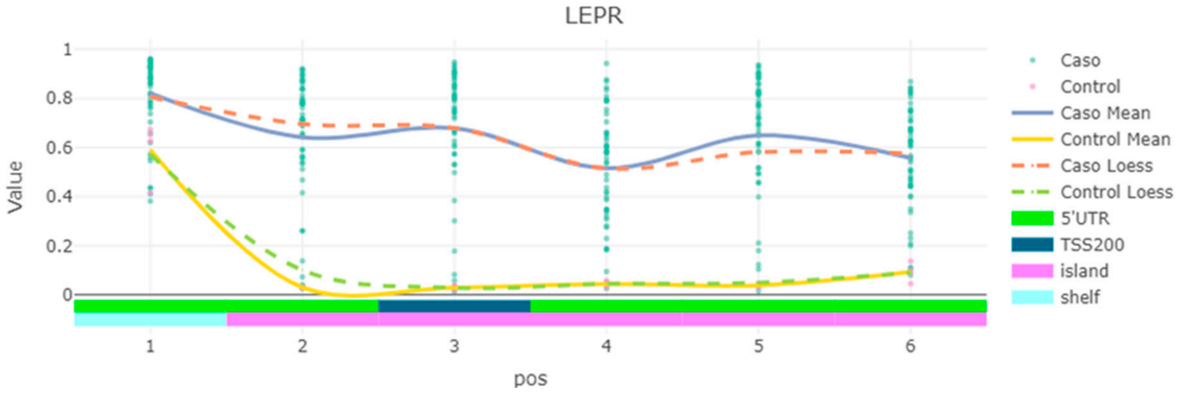

Supplementary Figure S4. DNA methylation profiles of selected genes with differentially methylated CpG sites. a) *BLK*, b) *BCR*, c) *ARID5B*, d) *MACROD2*, and e) *LEPR*. Each panel shows the methylation beta values across CpG sites in the indicated gene for ALL cases (yellow) and controls (blue). Solid lines represent individual samples, and dashed lines indicate loess smoothed curves for each group. The bottom tracks indicate genomic annotations for each CpG site: Gene regions: body (yellow), 5'UTR (green), first exon (light blue), TSS200 (dark blue), and TSS1500 (red). CpG context: island (pink), shore (green), shelf (light blue), and open sea (salmon). Genomic coordinates (probe positions) are shown on the x-axis.

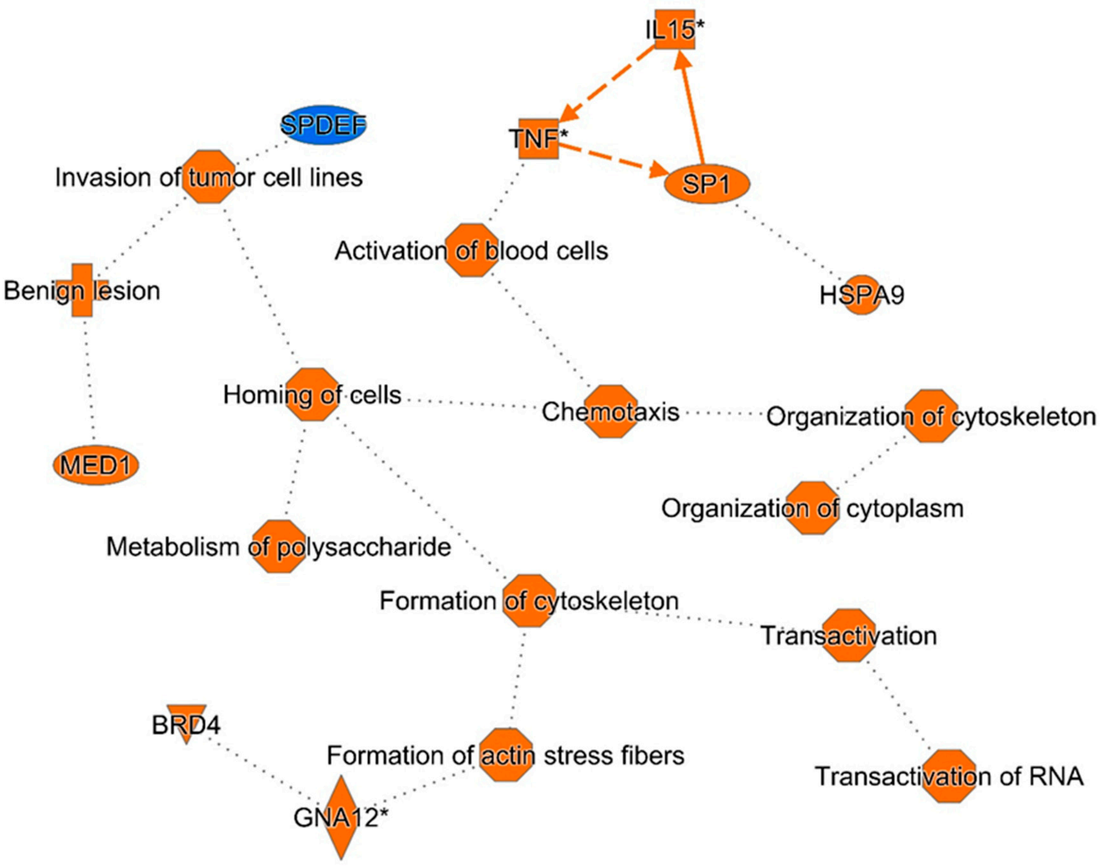

Supplementary Figure S5. Summary graph generated by Ingenuity Pathway Analysis (IPA) comparing ALL cases and controls. Orange symbols indicate activated functions or regulators, and blue symbols indicate predicted inhibition. The shape of each node denotes the type of molecule or function. Octagons: biological functions, ovals: transcription regulators, squares: cytokines or growth factors; diamonds: G-protein coupled receptors, triangles: transcription regulators with unknown or indirect roles. Solid lines indicate direct relationships, dashed lines indicate indirect relationships, and dotted lines represent predicted or less certain associations. * Molecules with statistical significance in activation or inhibition z-score predictions.

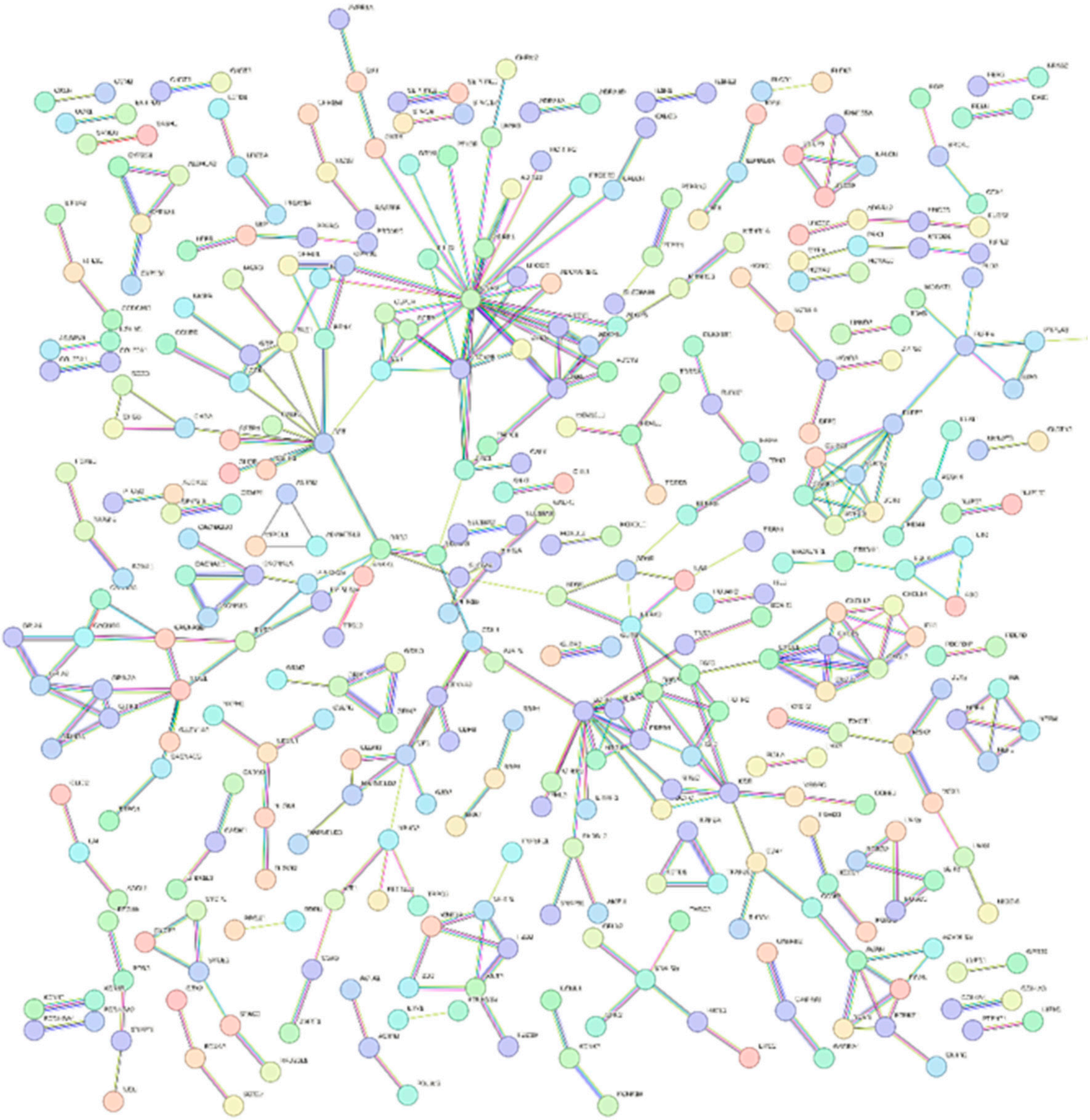

Supplementary Figure S6. Protein–protein interaction (PPI) network of differentially methylated genes. PPI network generated using STRING with a confidence score threshold of 0.9 and excluding proteins with no connections (disconnected nodes). The resulting network consists of 1,287 nodes and 374 edges. Edge thickness reflects the confidence of interaction evidence. The top four hub proteins with the highest degrees were GNAS (25 interactions), EGFR (14), SST (11), and ADCYAP1 (10), highlighted as central nodes in the network.

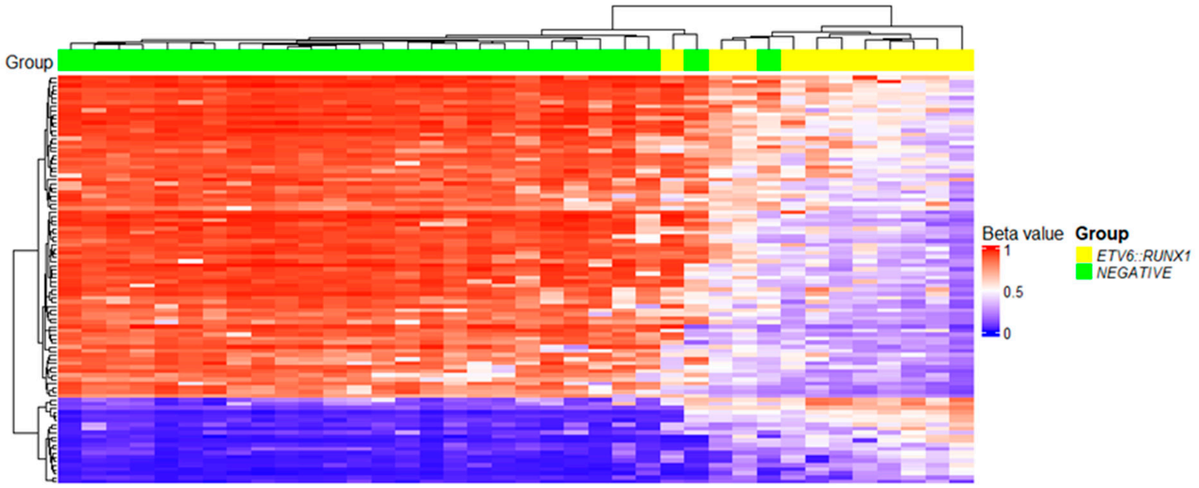

Supplementary Figure S7. Heatmap of the 100 most differentially methylated CpGs in *ETV6::RUNX1* ALL cases. Blue color background areas indicate highly methylated loci and red displays low methylated regions. In addition, the green, and gold colors above the heatmap indicate *ETV6::RUNX1* -positive and cases negative for *ETV6::RUNX1, TCF3::PBX1, BCR::ABL1,* and *KMT2A::AFF1* fusions.

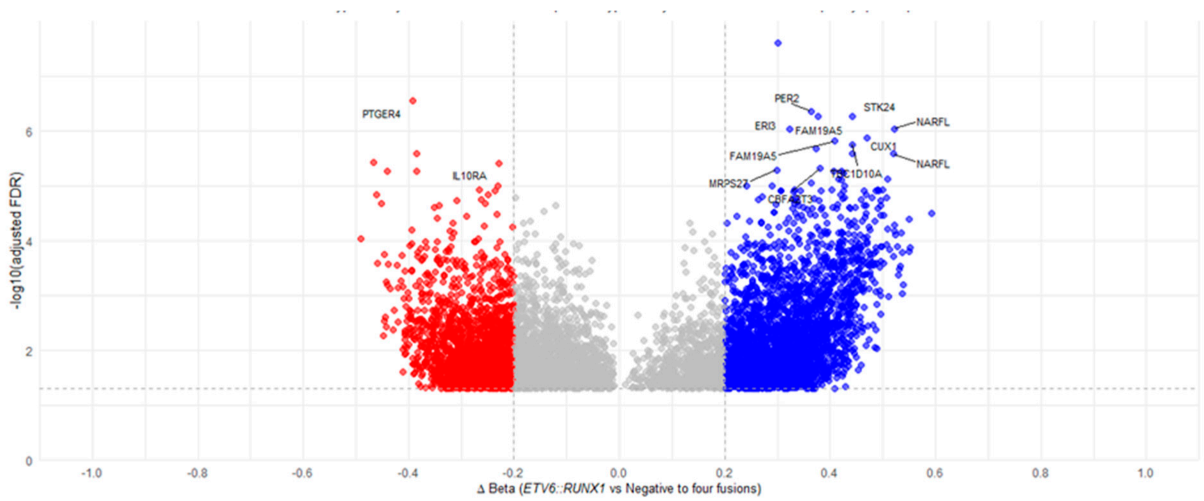

Supplementary Figure S8. Volcano plot of differentially methylated CpG sites between *ETV6::RUNX1*-positive and cases negative for *ETV6::RUNX1*, *TCF3::PBX1*, *BCR::ABL1*, and *KMT2A::AFF1* fusions. The x-axis shows the difference in methylation (Δβ), and the y-axis represents the –log10 of the adjusted p-value. Differentially methylated CpGs with FDR q-value < 0.05 and |Δβ| > 0.2 are presented. Colored dots represent hypermethylated (red) and hypomethylated (blue) probes that showed statistically significant differences between subgroups.

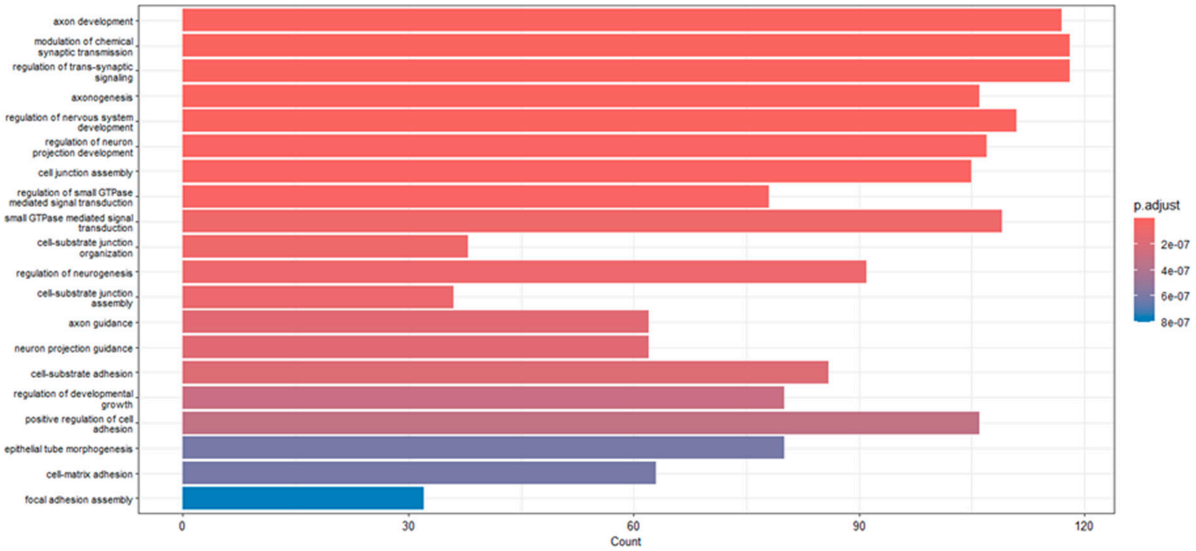

Supplementary Figure S9. Gene Ontology (GO) terms associated with *ETV6::RUNX1*. Top 20 enriched GO terms associated with genes related to the *ETV6::RUNX1* gene fusion. The analysis includes Biological Process, Molecular Function, and Cellular Component categories.

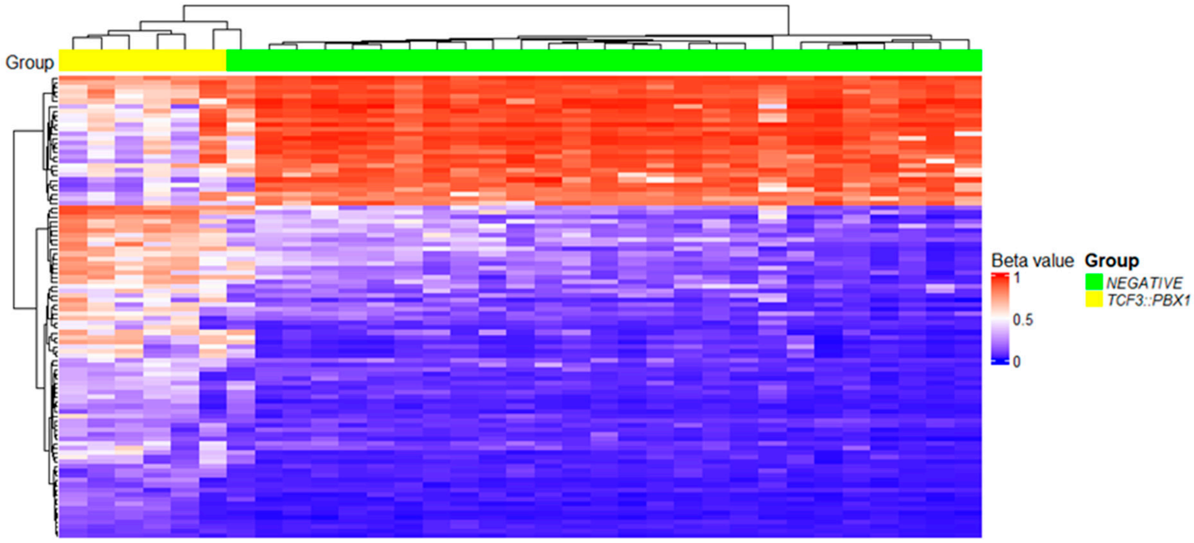

Supplementary Figure S10. Heatmap of the 50 most differentially methylated CpGs in *TCF3::PBX1* ALL cases. Blue color background areas indicate highly methylated loci and red displays low methylated regions. In addition, the purple, and orange colors above the heatmap indicate *TCF3::PBX1* -positive and cases negative for *ETV6::RUNX1*, *TCF3::PBX1*, *BCR::ABL1*, and *KMT2A::AFF1* fusions.

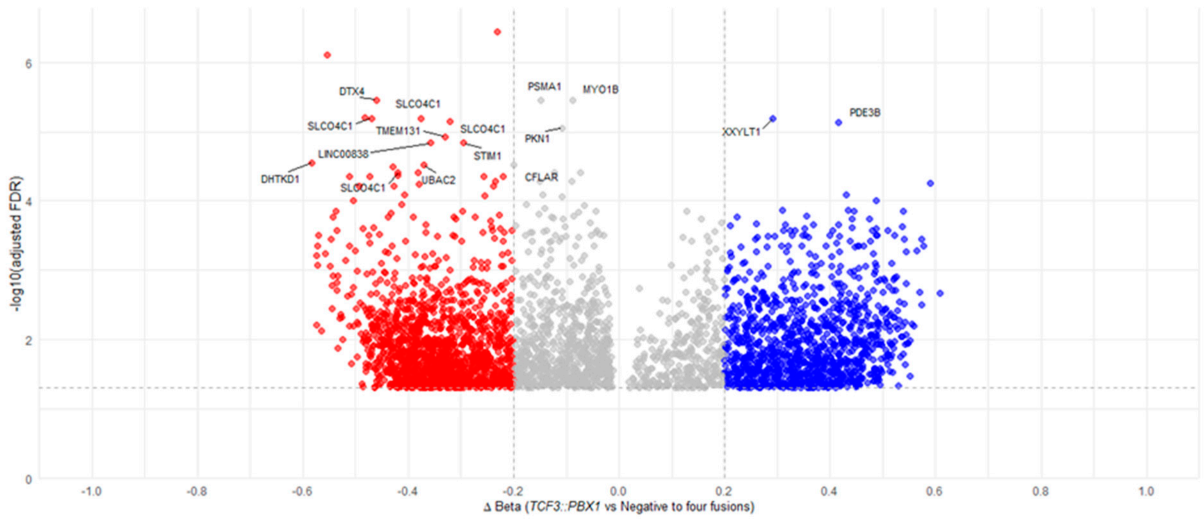

Supplementary Figure S11. Volcano plot of differentially methylated CpG sites between TCF3::PBX1-positive and cases negative. The x-axis shows the difference in methylation (Δβ), and the y-axis represents the –log10 of the adjusted p-value. Differentially methylated CpGs with FDR q-value < 0.05 and |Δβ| > 0.2 are presented. Colored dots represent hypermethylated (red) and hypomethylated (blue) probes that showed statistically significant differences between subgroups.

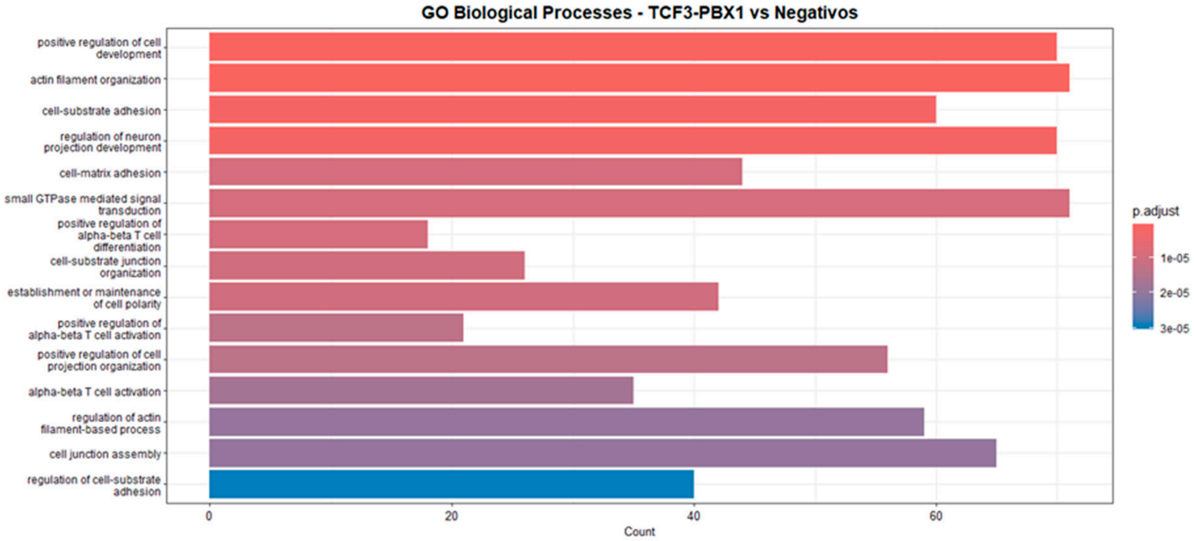

Supplementary Figure S12. GO terms associated with *TCF3::PBX1*. Top 20 enriched GO terms associated with genes related to the *ETV6::RUNX1* gene fusion. The analysis includes Biological Process, Molecular Function, and Cellular Component categories.

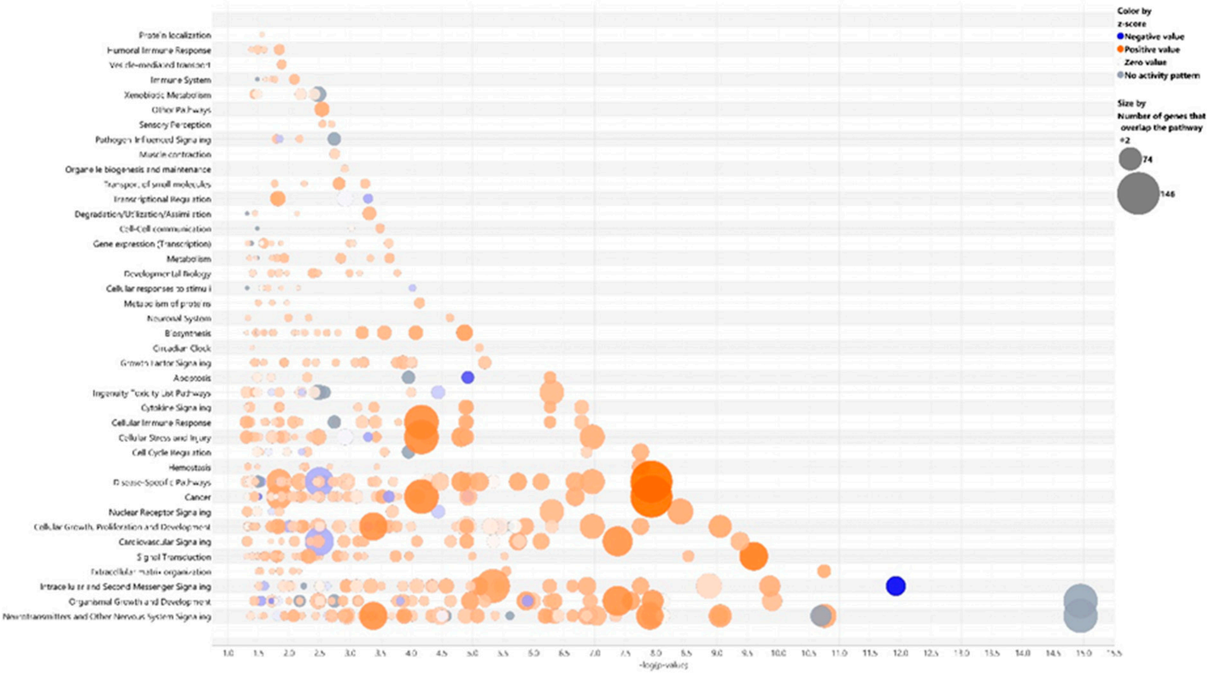

Supplementary Figure S13. Pathway analysis of altered biological pathways comparing early relapse ALL cases and no relapse ALL cases using a bubble plot. X-axis: Statistical significance ($-\log_{10}(p)$), where higher values indicate greater relevance. Bubble size represents the number of genes involved in each pathway. Color indicates the IPA (Ingenuity Pathway Analysis) z-score, which predicts the direction of regulation. Orange: significant activation (z-score > 2). Blue: significant inhibition (z-score < −2). Gray: statistically significant pathways ($p < 0.05$) with no predicted direction of change.
